# CalTrig: A GUI-Based Machine Learning Approach for Decoding Neuronal Calcium Transients in Freely Moving Rodents

**DOI:** 10.1523/ENEURO.0009-25.2025

**Published:** 2025-07-10

**Authors:** Michal A. Lange, Yingying Chen, Haoying Fu, Amith Korada, Changyong Guo, Yao-Ying Ma

**Affiliations:** ^1^Department of Biochemistry, Molecular Biology and Pharmacology, Indiana University School of Medicine, Indianapolis, Indiana 46202; ^2^Stark Neurosciences Research Institute, Indiana University School of Medicine, Indianapolis, Indiana 46202

**Keywords:** calcium transients, data visualization, GRU, in vivo calcium imaging, machine learning, miniScope

## Abstract

Advances in in vivo Ca^2+^ imaging using miniature microscopes have enabled researchers to study single-neuron activity in freely moving animals. Tools such as Minian and CalmAn have been developed to convert Ca^2+^ visual signals to numerical data, collectively referred to as CalV2N. However, substantial challenges remain in analyzing the large datasets generated by CalV2N, particularly in integrating data streams, evaluating CalV2N output quality, and reliably and efficiently identifying Ca^2+^ transients. In this study, we introduce CalTrig, an open-source graphical user interface (GUI) tool designed to address these challenges at the post-CalV2N stage of data processing collected from C57BL/6J mice. CalTrig integrates multiple data streams, including Ca^2+^ imaging, neuronal footprints, Ca^2+^ traces, and behavioral tracking, and offers capabilities for evaluating the quality of CalV2N outputs. It enables synchronized visualization and efficient Ca^2+^ transient identification. We evaluated four machine learning models (i.e., GRU, LSTM, Transformer, and Local Transformer) for Ca^2+^ transient detection. Our results indicate that the GRU model offers the highest predictability and computational efficiency, achieving stable performance across training sessions, different animals, and even among different brain regions. The integration of manual, parameter-based, and machine learning-based detection methods in CalTrig provides flexibility and accuracy for various research applications. The user-friendly interface and low computing demands of CalTrig make it accessible to neuroscientists without programming expertise. We further conclude that CalTrig enables deeper exploration of brain function, supports hypothesis generation about neuronal mechanisms, and opens new avenues for understanding neurological disorders and developing treatments.

## Significance Statement

Understanding neuronal activity at the single-cell level is critical for unraveling brain mechanisms underlying behavior and neurological disorders. CalTrig, a novel GUI-based tool, integrates machine learning with Ca^2+^ imaging analysis to address key challenges in processing complex, large-scale neural data from freely moving rodents. By providing synchronized visualization and flexible detection of Ca^2+^ transients, CalTrig enables researchers without programming expertise to extract meaningful insights into brain function. This tool's adaptability and computational efficiency support diverse research applications, from behavioral neuroscience to translational studies. In brief, CalTrig enhances the precision and scalability of Ca^2+^ transient analysis, paving the way for deeper exploration of neuronal dynamics and facilitating advancements in neuroscience research.

## Introduction

Neurons within the same brain region are diverse in type, connectivity, and activity, responding to stimuli with high temporal precision. One of the key advances in modern neuroscience is the shift from studying brain regions as functional units to focusing on individual neurons. Monitoring single-neuron activity in freely moving animals allows for deeper insights into brain function and dysfunction. Recent advances in in vivo imaging and fluorescent Ca^2+^ indicators, coupled with miniature microscopes (miniScope), have revolutionized the study of neural dynamics in freely moving animals. Tools have been developed to convert Ca^2+^ visual signals (i.e., Ca^2+^ imaging video files) to numerical data (e.g., changes in fluorescent signal intensity indicating the Ca^2+^ influx for each neuron), denoted CalV2N. Early tools, such as Suite2P (Pachitariu et al., 2016), SIMA (Kaifosh et al., 2014), STNeuronNet (Soltanian-Zadeh et al., 2019), and CalmAn (Giovannucci et al., 2019), were created to process two-photon imaging data. Due to the low resolution and poor signal-to-noise ratio (SNR) in one-photon imaging data, more specific CalV2N tools were created including MIN1PIPE (Lu et al., 2018) and Minian (Zhou et al., 2018). The algorithms used in CalV2N tools started with principal-component analysis/independent component analysis (PCA/ICA; Mukamel et al., 2009), and then upgraded to constrained non-negative matrix factorization (CNMF), and its various derivations such as CNMF-E (Zhou et al., 2018). Due to better reliability in demixing the activities of overlapping cells, the computational efficiency and accessibility of parameter adjustment, CNMF-based Minian has become a reliable choice and selected as the CalV2N tool to extract Ca^2+^ traces in this study.

Despite the availability of CalV2N tools, significant challenges remain in extracting meaningful insights from Ca^2+^ imaging data. Key issues include the lack of synchronized visualization for multiple data streams, inconsistent results due to global parameter application, and the uncertainty in verifying detected cells and Ca^2+^ traces. Current tools, like Minian, process data in discrete sections, limiting integrative analysis. Additionally, there are no efficient and reliable methods for identifying miniScope Ca^2+^ transients. These challenges are compounded by the complexity of the data, which feature high temporal (10–60 Hz) and spatial (0.8–1.0 µm pixel size) resolution, high cell throughput (50–200 neurons per animal), and the integration of behavioral tracking, resulting in vast, multifaceted datasets requiring advanced processing tools. To address these challenges at the post-CalV2N stage, we developed the Ca^2+^ transient identifier GUI (CalTrig), a graphical user interface (GUI) using the Python package PyQT5. CalTrig integrates all outputs from Minian (including Ca^2+^ imaging original processed videos, Ca^2+^ traces, cell footprints, and behavioral videos) to facilitate synchronized visualization, evaluate the performance of CalV2N, and identify Ca^2+^ transients.

**Figure 1.**
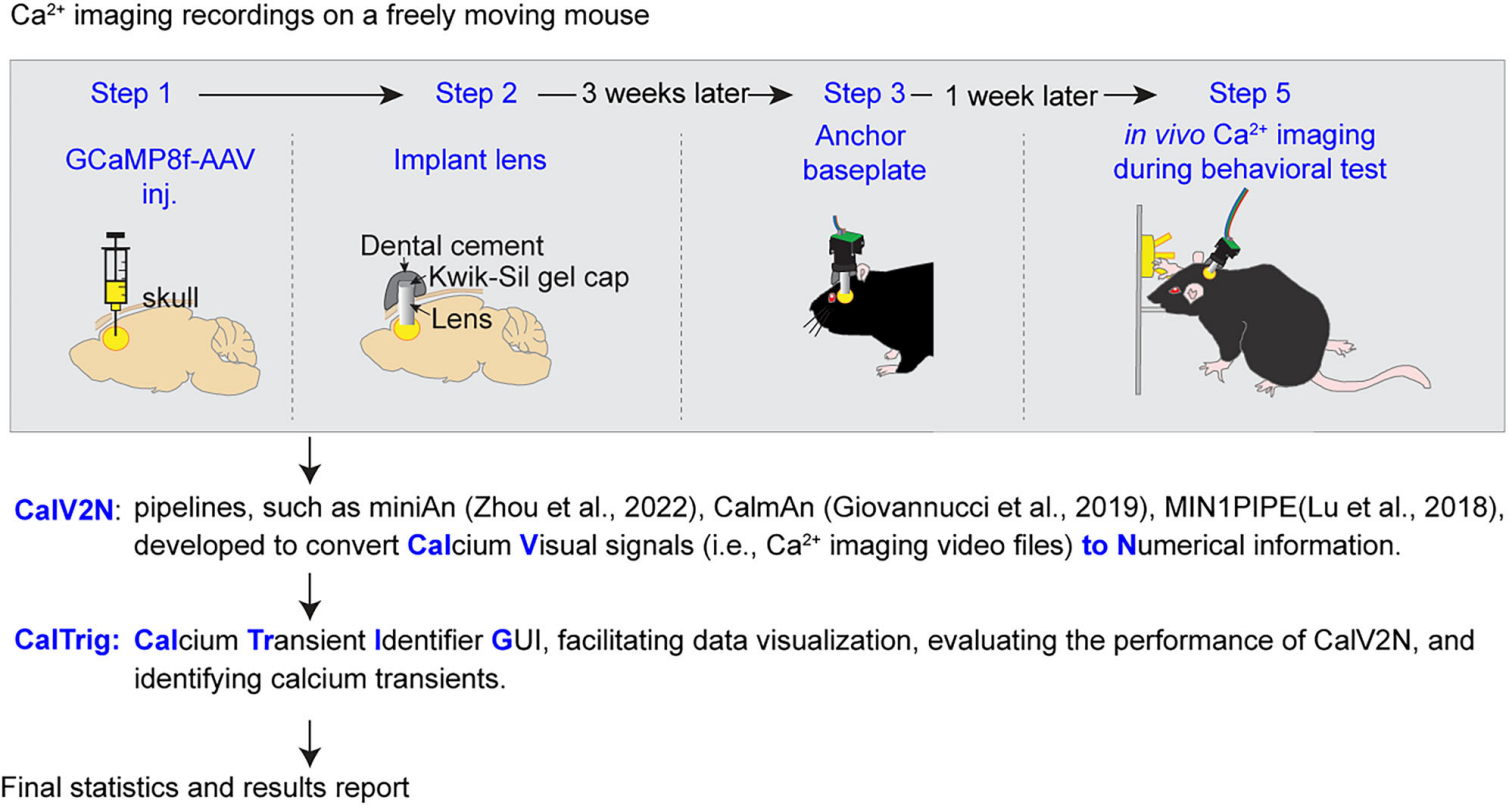
Flowchart showing the procedures of an in vivo Ca^2+^ study and highlighting the role of CalTrig in addressing gaps in data processing and analysis.

In summary, CalTrig is developed to bridge the gap between CalV2N tools and final analysis stages, streamlining workflows and extracting meaningful biological insights as illustrated in Figure 1. Although the potential readership is broad, this research article primarily aims to assist neurobiologists in processing in vivo Ca^2+^ imaging data collected using a single-photon miniScope. We would like to give a brief introduction to the article's organization, which differs from a typical neurobiological research paper. The Materials and Methods section not only reiterates previously established procedures for collecting Ca^2+^ imaging data from freely moving mice and extracting the Ca^2+^ traces, as we reported earlier (Fang et al., 2023; Chen et al., 2024), but also provides detailed information about the newly developed tool, CalTrig. The latter includes data loading from CalV2N, data visualization, cell verification, CalV2N evaluation, Ca^2+^ transient identification, and exporting figures or data for statistical analysis, ready for publication. We present three strategies for Ca^2+^ transient identification (i.e., parameter-based, manual, and machine learning-based detection), including their procedures and applications. In the Results section, we compare the performance of multiple machine learning models, evaluate whether the established machine learning model can be used to identify transients across datasets collected at different time points, from different animals, and in various brain regions. Finally, the Discussion highlights the advantages of using machine learning models, the integrative visual exploration interface, and the unique features of CalTrig. A list of key abbreviations is provided in Table 1 to facilitate readability.

## Materials and Methods

### In vivo Ca^2+^ imaging data collection

#### Experimental animals

All in vivo procedures on laboratory animals were performed in accordance with the United States Public Health Service Guide for Care and Use of Laboratory Animals and were approved by the Institutional Animal Care and Use Committee at Indiana University School of Medicine. Ten male C57BL/6J mice, bred in-house using breeders originally derived from the Jackson Laboratory, were used in this study. The mice were group housed, except for those with GRIN lens implants, which were singly housed to prevent cage mates from damaging the implanted lens. All mice had *ad libitum* access to chow and water in home cage, maintained on a 12 h light/dark cycle (light on at 7:00 A.M. and off at 7:00 P.M.).

#### Surgical procedures

##### Microinjection of AAV

Mice were anesthetized with 2.5% isoflurane for induction and maintained with ∼1.2%. A 28-gauge injection needle was used to unilaterally inject the AAV1-Syn-jGCaMP8f-WPRE or AAV1-CaMKIIa-jGCaMP8f-WPRE solution (0.5 µl/site, 0.1 µl/min) via a Hamilton syringe into the secondary motor cortex (M2; coordinates in mm: AP, +1.80; ML, ±0.60; DV, −1.30) or the medial prefrontal cortex (mPFC; coordinates in mm: AP, +2.05; ML, ±0.3; DV, −2.45) using a Pump 11 Elite Syringe Pumps (Harvard Apparatus). Injection needles were left in place for 5 min following injection.

##### Lens implantation

A couple of minutes after withdrawing the AAV injection needle, a unilateral Gradient Refractive Index (GRIN) lens (Inscopix, #1050-004595; diameter, 1.0 mm; length, ∼4.0 mm; working distance, 200 µm) was lowered through the cranial window to 200 µm above the center of the virus injection site. The open space between the lens' side and the skull opening was sealed with surgical silicone (Kwik-Sil) and secured by dental cement (C&B Metabond). The exposed part of the lens above the skull was further coated with black cement (Lang Dental).

##### Base plating

Three weeks later, mice were anesthetized with isoflurane again. The cement on top of the GRIN lens was carefully removed using drill bits until the lens was exposed. The top of the lens was then cleaned using lens paper and a cleaning solution. A metal baseplate was mounted onto the skull over the lens using Loctite super glue gel, guided by a MiniScope V4.4 (Open Ephys) for optimal field of view. Once the baseplate was securely mounted, the MiniScope was removed. A protective cap was attached to the baseplate, and mice were returned to home cage.

##### Verification of AAV expression and the lens location

After the completion of the in vivo Ca^2+^ imaging, M2 or mPFC-containing coronal slices were prepared as described previously (Huang and Ma, 2023) and then fixed in 4% paraformaldehyde for no less than a couple of hours. After a brief rinse with Phosphate-Buffered Saline, slices were mounted with ProLong Gold antifade mounting reagent with DAPI (Invitrogen, catalog #P36931). Confocal imaging was performed using a Zeiss LSM 800 confocal microscope. The criteria for animal inclusion in this study were (1) highly enriched AAV expression in mostly pyramidal neurons, within-M2 or mPFC viral injection site, and (2) the footprint of the GRIN lens tip at the top of the targeting brain area.

#### In vivo Ca^2+^ imaging recording

Mice were habituated to the in vivo Ca^2+^ recording procedure by mounting the miniScope V4.4 (Open Ephys) to the preanchored baseplate and recording for 5 min per day at the home cage for 3 d before starting the 1 h daily recordings in an operant chamber (Med Associates). Data Acquisition box, supported by an open-source, C^++^ and Open Computer Vision libraries-based software, was used to collect both Ca^2+^ and behavioral video streams simultaneously controlled by the operant chamber software, MED-PC (Med Associates) via a TTL adaptor. The sampling frequency was 30 Hz.

### Extraction of Ca^2+^ transient traces from the raw videos

Among multiple types of computational tools established previously to extract Ca^2+^ transients from raw videos, a Python-based analysis pipeline, Minian (Dong et al., 2021), was used in our data analyses due to its low memory requirement and user-friendly parameter options. In brief, there were five steps in the pipeline. First, multiple raw videos were batch loaded and subjected to a PREPROCESSING stage, where sensor noise and background fluorescence from scattered light were removed. Second, rigid brain motions were corrected by MOTION CORRECTION. Third, the initial spatial and temporal matrices for later steps were generated by a seed-based approach, called SEEDS INITIALIZATION. Fourth, the spatial footprints of cells were further refined. Fifth, the temporal signals of cells were also refined. The last two steps, the SPATIAL UPDATE, and the TEMPORAL UPDATE, as the core computational components based on CNMF algorithm, were repeated at least one more time.

### CalTrig (Ca^2+^ transient identification GUI)

CalTrig is a Python-based open-source code with GUI interface. The repository contains documentation, demos, and a message/discussion board. The code, which is compatible with Python 3.10, uses several open-source libraries including Xarray, Numpy, Pandas, PyQt5, and Pyqtgraph.

Computer system specifications for CalTrig development and testing: The development and testing of CalTrig were conducted on a system with the following specifications: CPU, Ryzen 9 7900X; RAM, 64GB; GPU, Nvidia RTX 4080; Operating System, Windows 11.

#### Data loading

**Figure 2.**
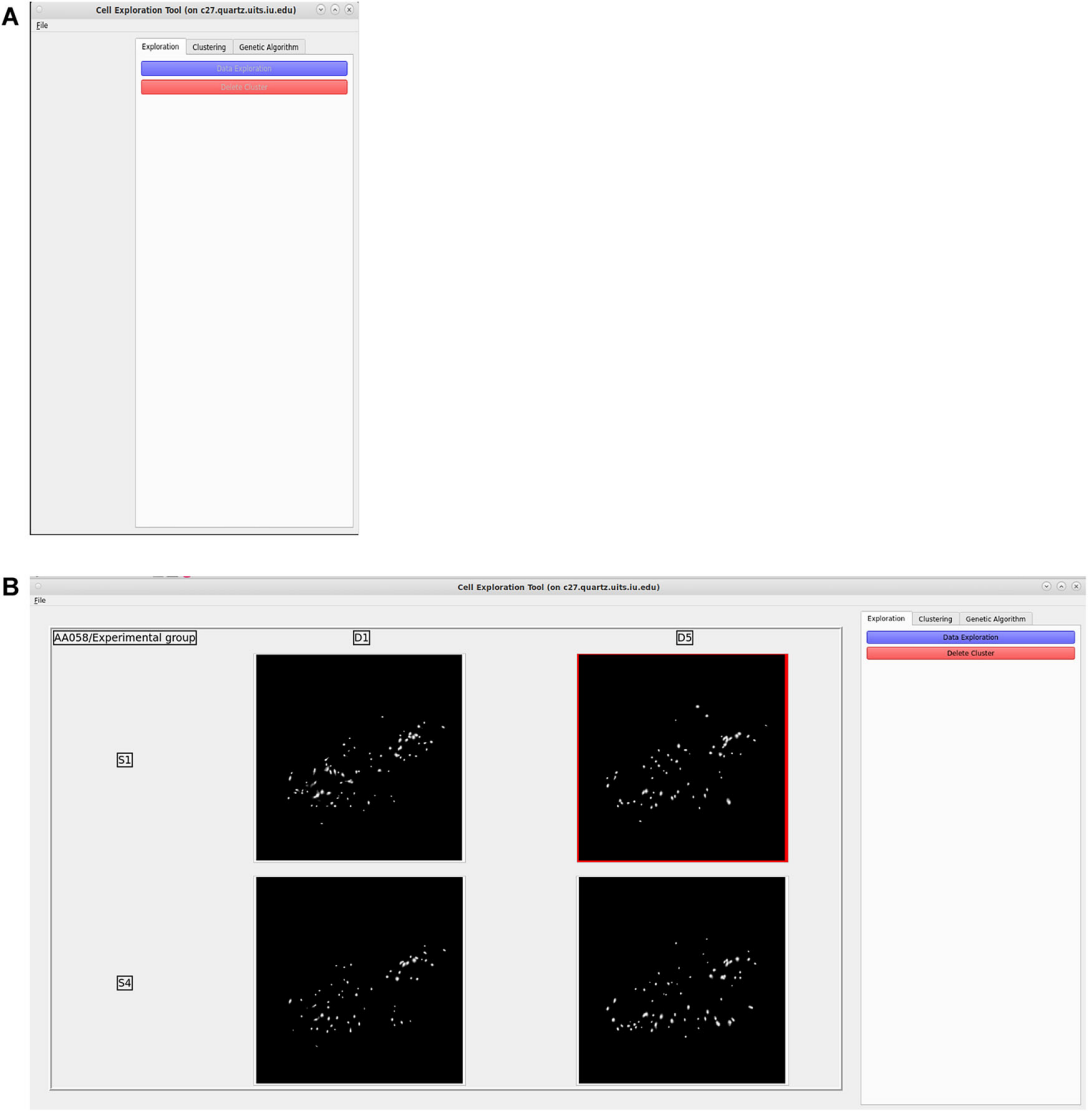
Data loading. ***A***, The front page of CalTrig serves as a hub for loading data. ***B***, Each set of data can be visualized in the window by their cell footprint and are arranged in a grid related to the experiment design details, such as animal ID, day, and session stage.

The front page of CalTrig serves as a hub for loading data (Fig. 2*A*). Data can be loaded by directly specifying the file path, or indirectly by loading the pregenerated INI (Initialization) files or JSON (JavaScript Object Notation) files.

**Figure 3.**
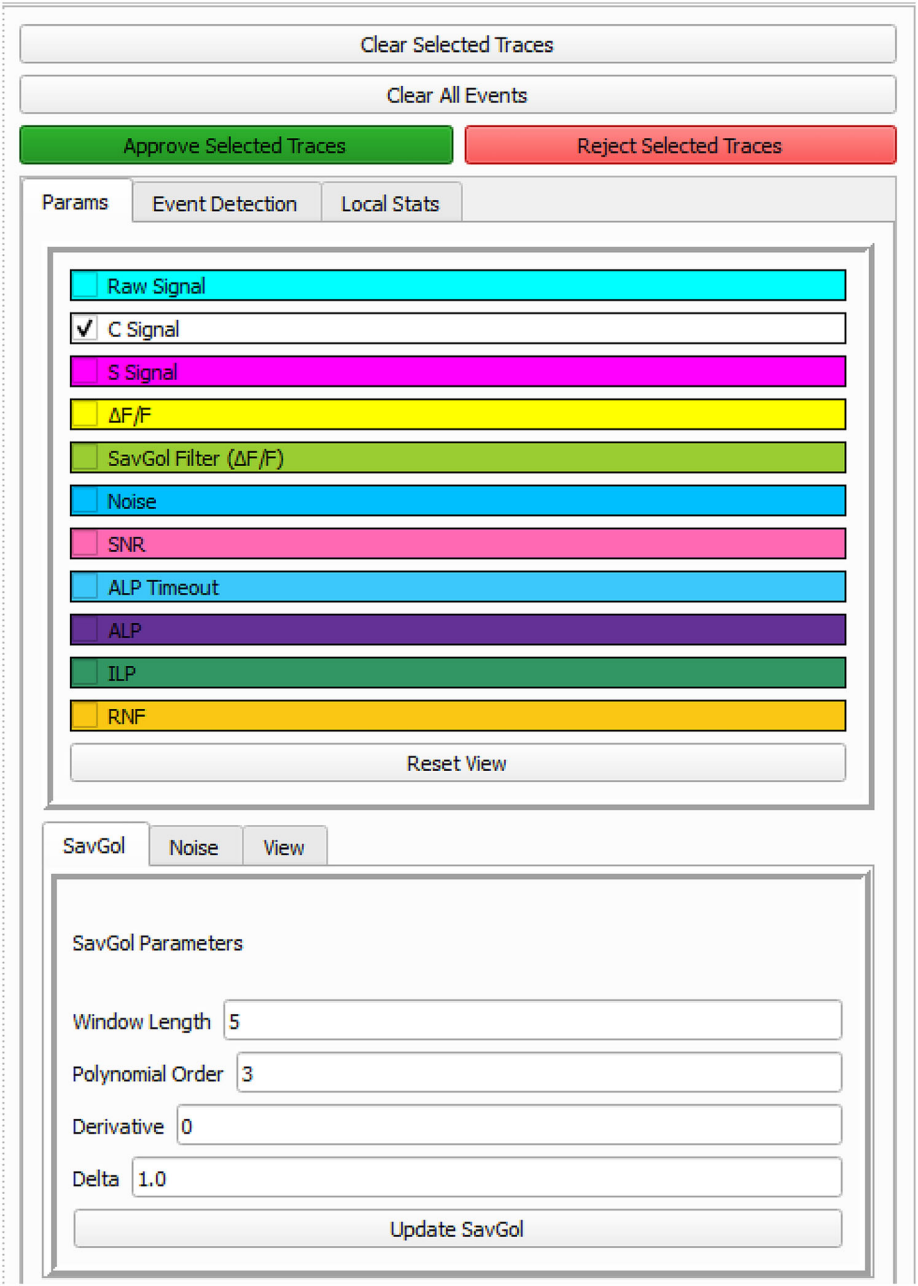
Parameter list. In top panel of the Trace Toolbox, raw signal, C signal, S signal, Δ*F*/*F*, Noise, Signal to noise ratio (SNR), as well as other behavioral readouts (e.g., active lever press, ALP; ALP during timeout; inactive lever press; and reinforcement, RNF) are listed as items to be selected to show in the Ca^2+^ trace window. The parameters of Savitzky–Golay filter (SavGol), used to estimate the SNR, are listed in the bottom panel.

The INI file uses a flat format with itemized information to detail a single dataset. It incorporates both basic experimental design details (e.g., Animal ID, day, session stage) and multiple temporally synchronized data lines, including (1) the original and CNMF processed Ca^2+^ image video; (2) extracted Ca^2+^ trace data (i.e., the CNMF output); (3) behavioral data (i.e., operant behavioral such as active lever press, ALP; inactive level press, ILP; and Reinforcement, RNF); and (4) behavioral video tracking. The variables included in each CNMF dataset are listed in Table 2 and included in Parameter list in CalTrig interface (Fig. 3). The INI file can be created by following the format provided in demo INI files, ensuring that both the experimental design and data streams are properly synchronized for exploration and analysis.

**Table 1.** Key abbreviations

Abbreviation	Full spelling
ALP	Active lever press
CalTrig	Ca^2+^ transient identifier GUI
CalV2N	Ca^2+^ visual signals to numerical
CNMF	Constrained non-negative matrix factorization
dfRegion::dfMouse	A different brain region in a different mouse
dfSS::dfDay	A different session stage in a different daily session
dfSS::smDay	A different session stage in the same daily session
FN	False negative
FP	False positive
GRIN	Gradient Refractive Index
GRU	Gated recurrent unit
GUI	Graphical user interface
ILP	Inactive level press
ITI	Inter-transient interval
LSTM	Long short-term memory
M2	Supplementary motor cortex
mPFC	Medial prefrontal cortex
PCA/ICA	Principal-component analysis/independent Component analysis
RNF	Reinforcement
RNN	Recurrent Neural Networks
smRegion::dfMouse	The same brain region in a different mouse
smSS::dfDay	The same session stage in a different daily session
smSS::smDay	The same session stage in the same daily session
SNR	Signal-to-noise ratio
TN	True negative
TP	True positive

**Table 2.** CNMF variable

CNMF variable	Definition
C	Temporal matrix of Ca^2+^ dynamics
S	Temporal matrix of Ca^2+^ transient rising slope
A	Spatial matrix of cell footprints
B	Spatial matrix of background footprints
F	Temporal vector of background dynamic.
YrA	Temporal matrix of residual cell traces.
Y/….	A collection of 3D matrices containing original and processed videos.
Δ*F*/*F*	Temporal matrix of Δ*F*/*F* values for cells. This is the only variable that is not considered mandatory, as the Minian pipeline does not generate it. When absent, it will be automatically generated in the CalTrig using a CAIMAN implementation.

The JSON file, in contrast, uses a hierarchical format, representing the experimental design. Using data from one of our addiction projects as an example, the JSON file can collect information from multiple INI files, outlining details such as treatments for each animal, Ca^2+^ imaging days, and session stages. The JSON file can be generated by saving data after loading multiple INI files, either for longitudinal recordings in a single animal or for a group of animals assigned to the same experimental group.

#### Data visualization

**Figure 4.**
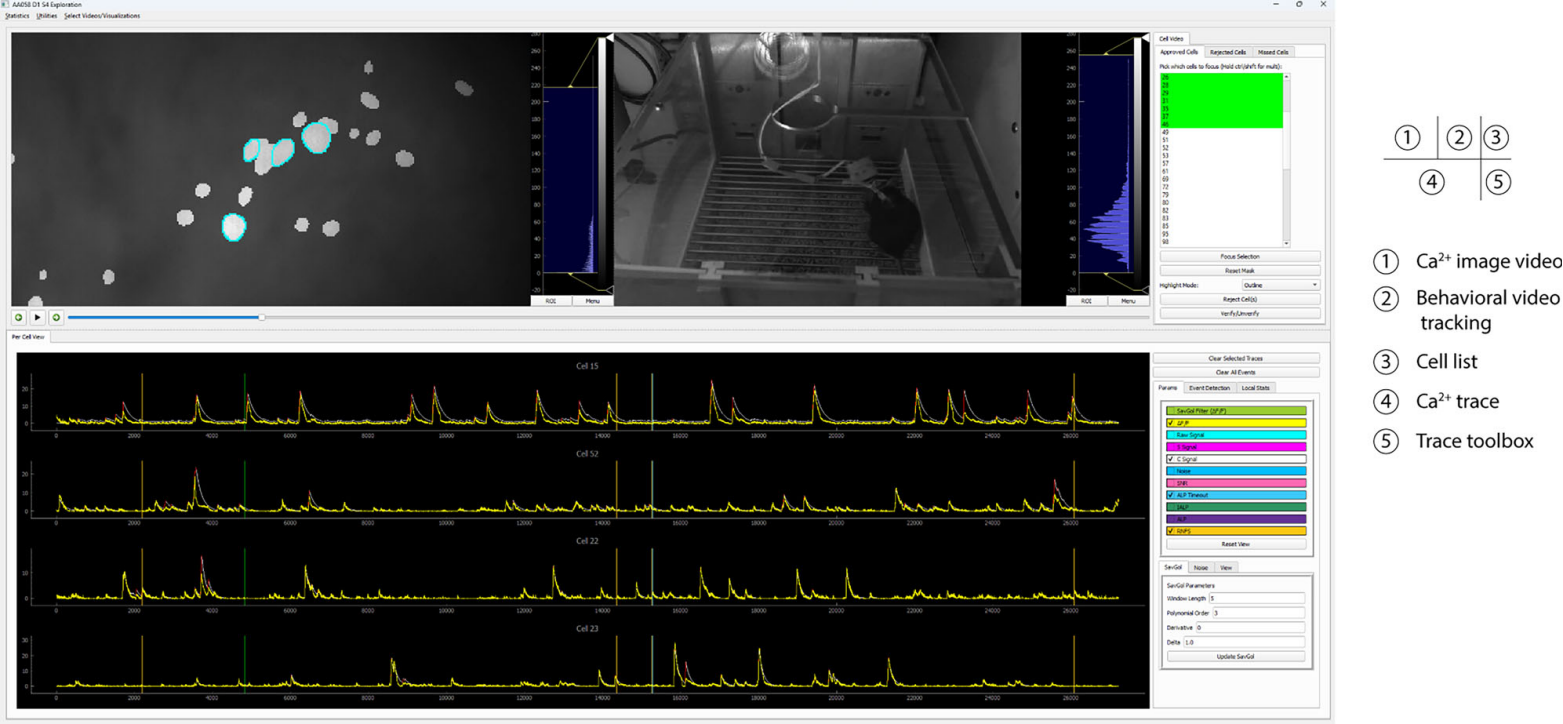
Five windows in CalTrig interface.

As shown in Figure 2*B*, each set of data can be visualized in the window by their cell footprint and are arranged in a grid related to the experiment design details, such as animal ID, day, and session stage. Select the dataset to be explored by clicking the corresponding window of the footprint, and then click the “cell exploration” button to open a separate window that is subdivided into five main components (Fig. 4), including the following:

1. Ca^2+^ imaging video: toggle between original versus CNMF processed videos can be zoomed in to see more details about the cellular signal.
2. Behavioral video tracking, showing behaviors of the freely moving animal during Ca^2+^ imaging recording.
3. Cell list, including individual cell number identified by CNMF.
4. Ca^2+^ trace window.
5. Trace toolbox.

**Figure 5.**
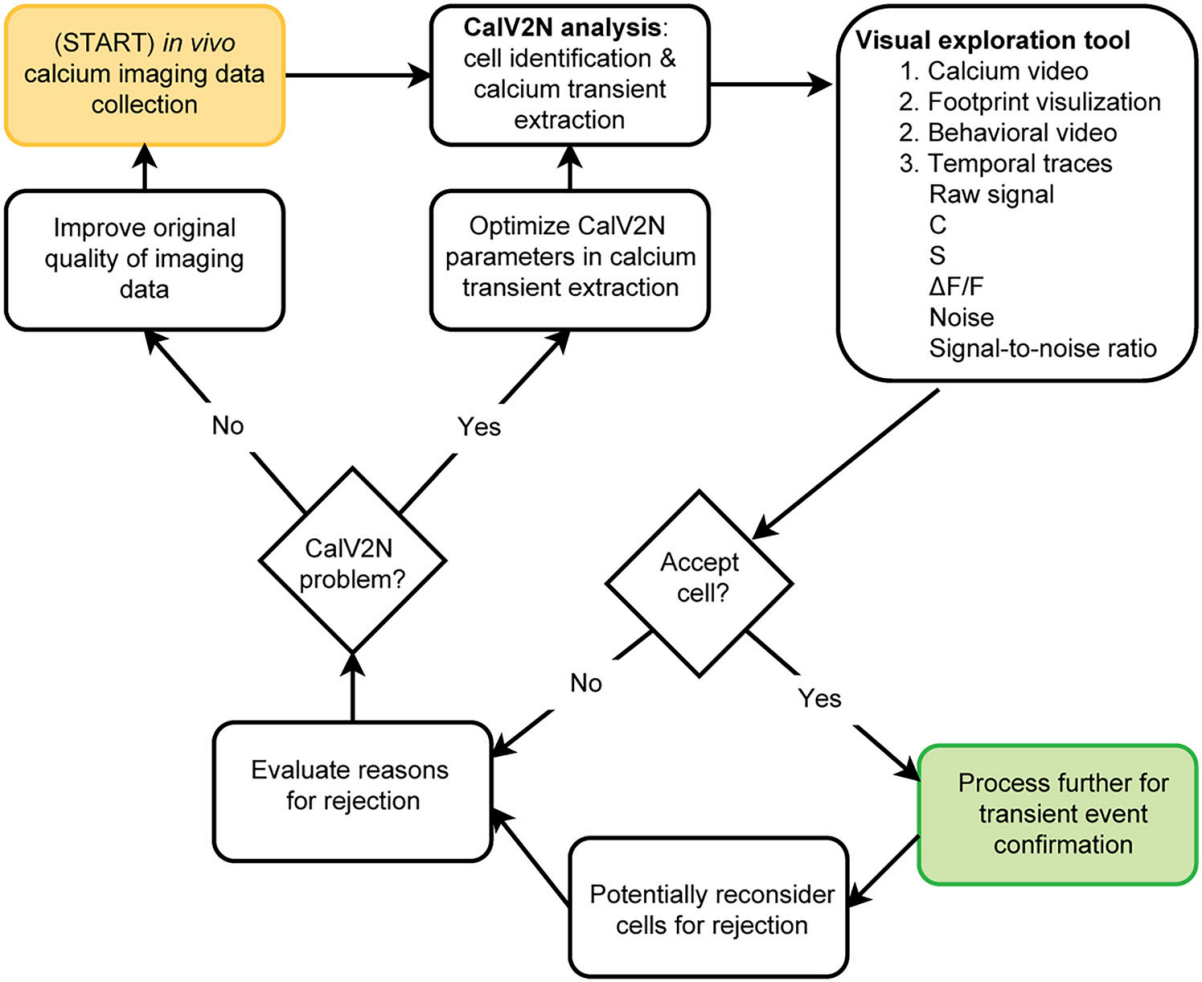
Use CalTrig to validate and filter cells identified by CalV2N. This flowchart illustrates the workflow for validating and filtering cells detected by CalV2N using CalTrig. The process begins with in vivo calcium imaging data collection, followed by CalV2N analysis, which performs cell identification and calcium transient extraction. To assess the quality of detected cells, a visual exploration tool is used, allowing interactive inspection of calcium video, footprint visualization, behavioral video, and temporal traces, including raw signal, C, S, Δ*F*/*F*, noise, and signal-to-noise ratio. Users determine whether to accept or reject a detected cell based on visual confirmation of its footprint, calcium image, and calcium transients. If a cell is accepted, it proceeds to further processing for transient event confirmation. If rejected, the reasons for rejection are evaluated, which may include poor signal quality, suspicious footprint, or statistical outliers. The rejection process also considers whether the issue stems from suboptimal CalV2N performance, in which case parameters for calcium transient extraction can be optimized. If CalV2N errors persist, improvements in raw imaging data quality should be considered. See more details in Materials and Methods, Cell verification and CalV2N evaluation.

The following tasks can be done by the cross talk between windows:

1. Footprint of cells of interest: The footprint of the detected cells can be visualized by (1) clicking on the projection area of a specific cell or selecting an image region containing multiple cells in the Ca^2+^ imaging video or (2) choosing the cell number from the cell list. This action superimposes its corresponding footprint onto the Ca^2+^ imaging video. The footprint can be displayed as a solid patch, a contour or by dimming the noncell intensity.
2. Ca^2+^ traces of cells of interest: Display the Ca^2+^ transient traces of the cells of interest, which can be selected by clicking on the footprint area in Ca^2+^ imaging video, or selection of the cell number in the cell list.
3. Variable readouts of Ca^2+^ traces: Ca^2+^ traces can be visualized with different temporal readouts as listed in Trace toolbox, including the variables directly imported from CNMF (i.e., C signal and S signal), the variables calculated by CalTrig (i.e., Δ*F*/*F*, raw signal, SavGol filter of Δ*F*/*F*, noise, SNR).
4. Time stamp of behavioral events or external stimuli on Ca^2+^ traces: Ca^2+^ traces can be segmented by the behavioral readout listed in the Trace toolbox, such as ALP, ILP, and RNF.
5. Standardized window size of the Ca^2+^ trace window: The window size for the *x*-axis in the unit of frame, and the scale range for the *y*-axis, can be defined in this box. When clicking “Reset view” button, the magnification of the signal is adjusted according to the *y-*axis range, and the window is set up with the predefined size by anchoring the first visible frame in the Ca^2+^ trace window. This is considerably helpful when manually identifying or verifying Ca^2+^ transients.
6. Cell verification: Cells identified by CNMF can be individually reviewed and categorized into “Approved cells,” “Rejected Cells,” and Missed Cells” as detailed below by cross talk between multiple windows and illustrated in Figure 5.
7. Quality evaluation of CNMF analysis: CalTrig allows user to verify detected cells, manually identify missing cells, and refine transient detection (see details below, Cell verification and CalV2N evaluation).
8. Ca^2+^ transient identification: this can be done by parameter-based autodetection, manual detection, machine learning-based detection, or combination of multiple strategies we have developed in CalTrig.

#### Cell verification and CalV2N evaluation

##### Cell verification.

**Figure 6.**
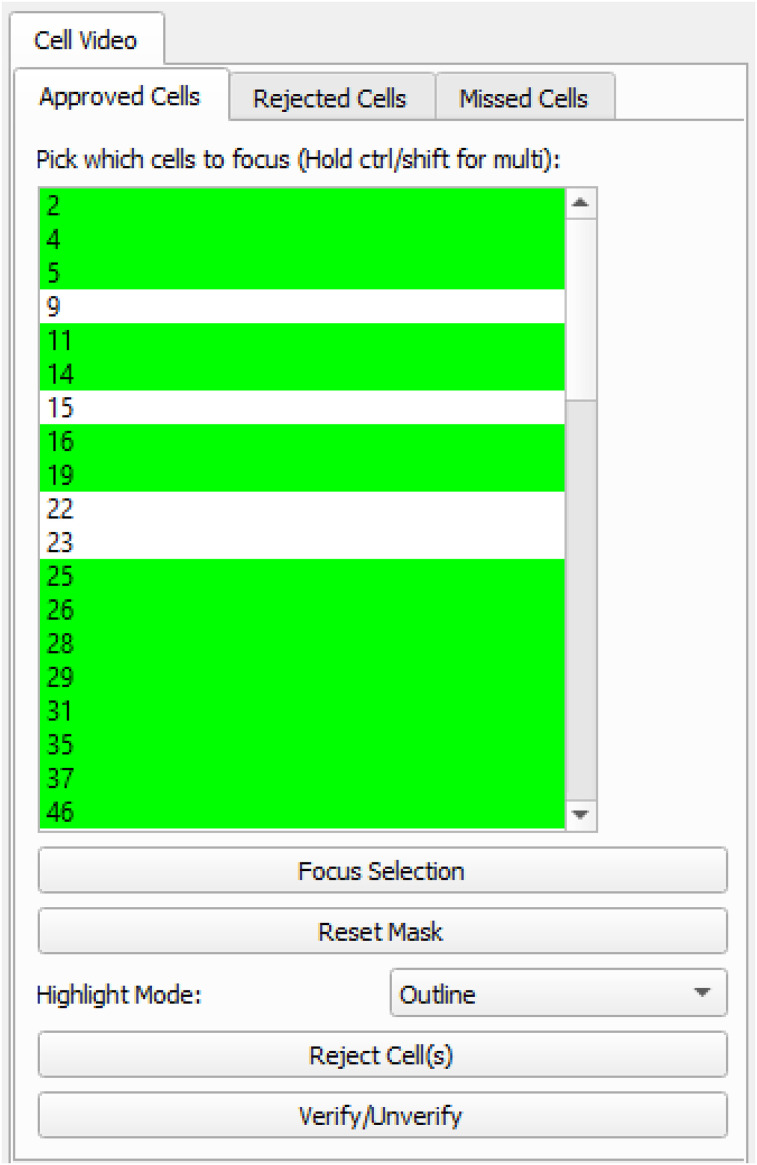
Cell verification. CalTrig allows user to inspect individual cells detected by CalV2N by interactive exploration of the Ca^2+^ imaging window and Ca^2+^ trace window. A cell becomes verified after a visual confirmation of its footprint, Ca^2+^ image, and Ca^2+^ trace, ensuring identifiable Ca^2+^ transients are present. The verified cells are highlighted in green.

**Figure 7.**
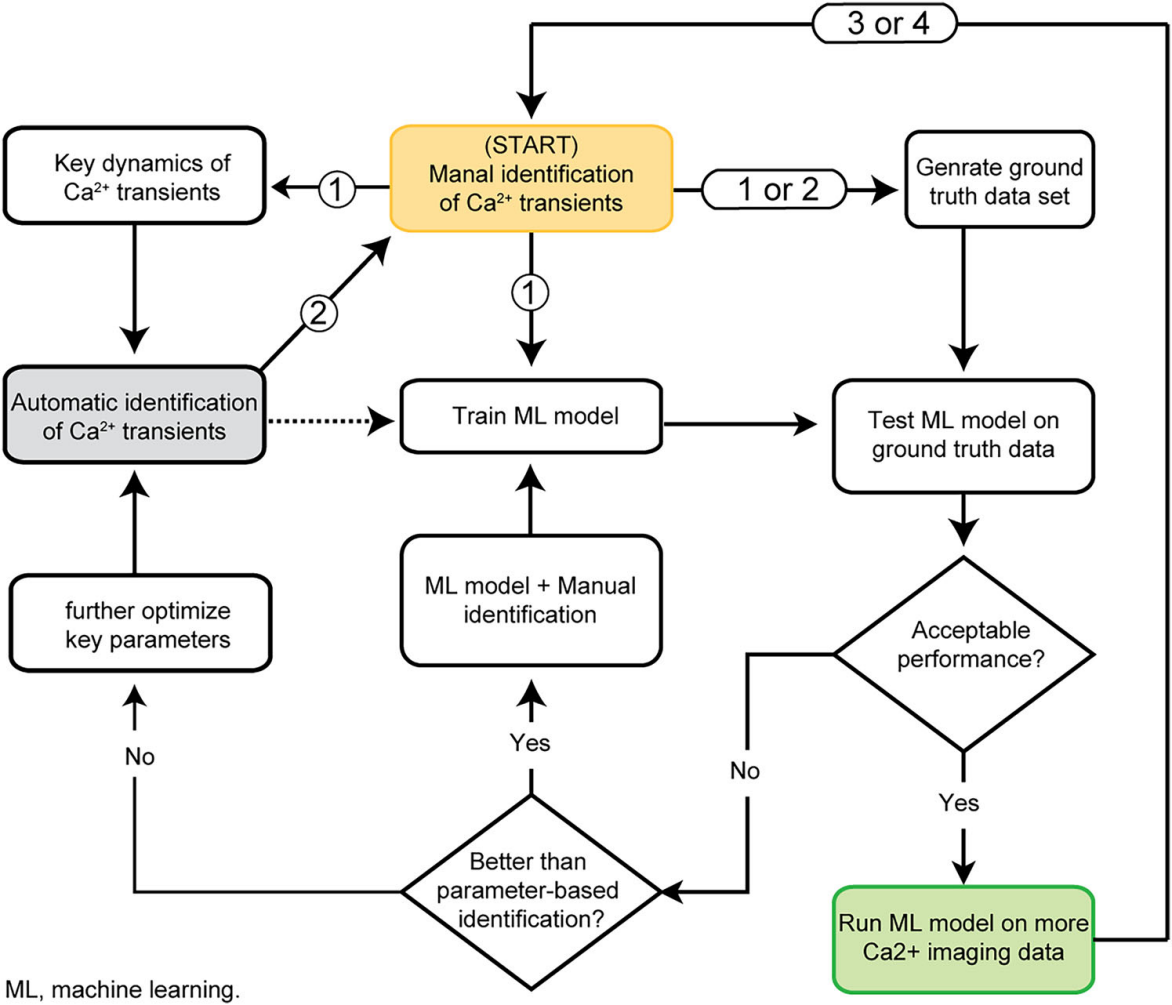
Use CalTrig to identify Ca^2+^ transients through an integrative strategy that combines manual detection, parameter-based autodetection, and machine learning-based detection. Circled numbers (1–4) mark the corresponding steps for the four applications discussed in the main text.

**Figure 8.**
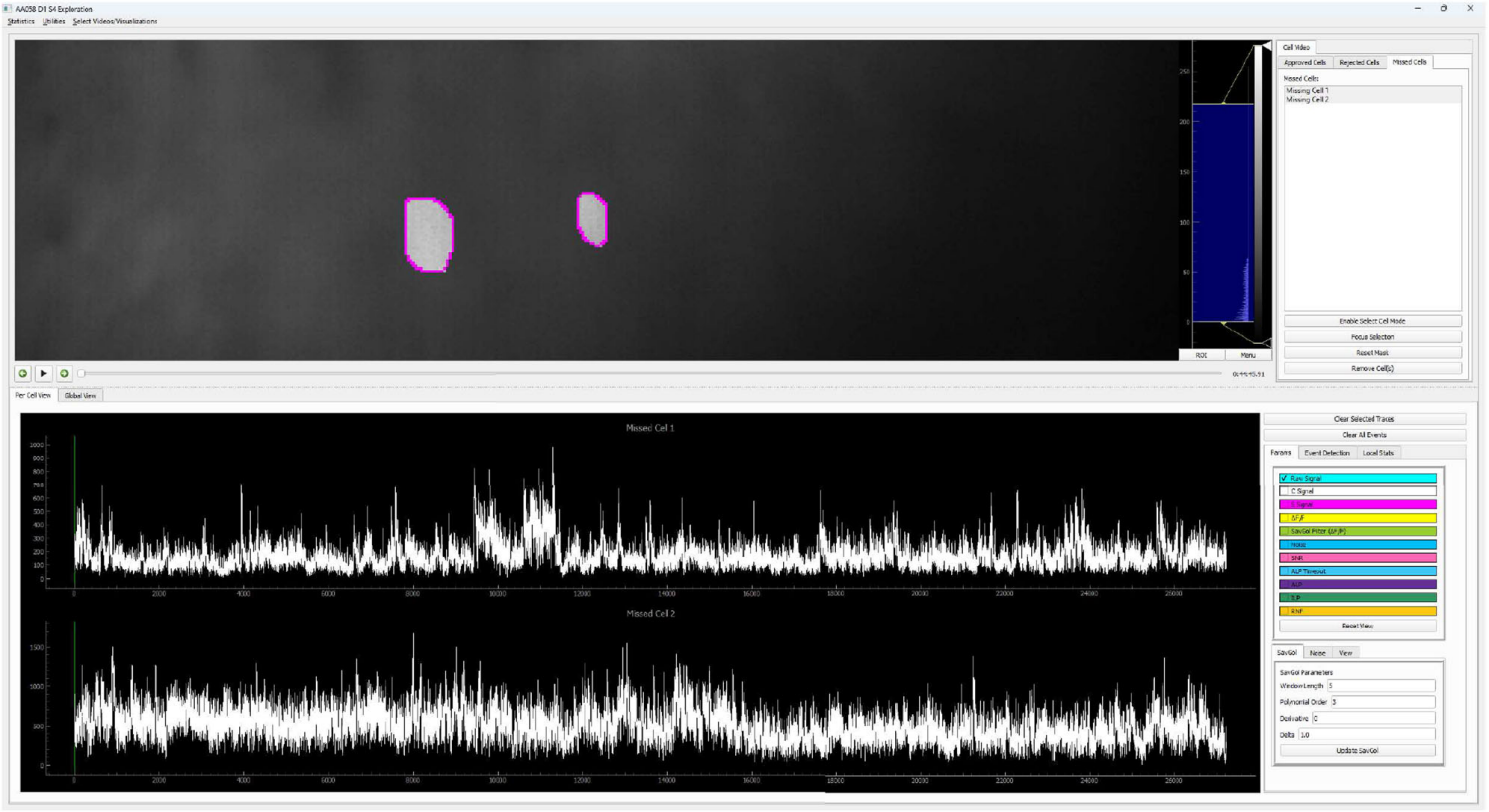
Add missing cell. While reviewing the Ca^2+^ imaging video, one may notice a potentially missed cell by CalV2N. CalTrig allows user to manually draw the contour of the suspected cell in the Ca^2+^ imaging window, creating its footprint. The corresponding temporal trace of signal intensity for the selected area is then calculated by averaging pixel intensities and displayed in the Ca^2+^ trace window. If the Ca^2+^ traces meet the criteria for accepted cells, the manually identified cell can be added to the list of missing cells.

- Verified Cell: CalTrig allows user to inspect individual cells detected by CalV2N by interactive exploration of the Ca^2+^ imaging window and Ca^2+^ trace window. A cell becomes verified after a visual confirmation of its footprint, Ca^2+^ image, and Ca^2+^ trace, ensuring identifiable Ca^2+^ transients are present (Fig. 6). Once verified, the cell can be analyzed using tools developed for Ca^2+^ transient identification, as outlined in Figure 7.
- Rejected Cell: Cells can be assigned to the rejection column for different reasons, including no identifiable Ca^2+^ transients, suspicious footprint, statistically identified as an outlier of the detected Ca^2+^ transients, etc. CalTrig provides the option to provide a written justification for each cell rejection, which can be used to provide feedback and potentially rerun the CNMF pipeline.
- Missing Cell: Automatic time-series-based algorithms may not be sensitive enough to detect cells with low to medium SNR, or those constitutively active or inactive with minimal dynamic changes. While reviewing the Ca^2+^ imaging video, one may notice a potentially missed cell by CalV2N. CalTrig allows user to manually draw the contour of the suspected cell in the Ca^2+^ imaging window, creating its footprint (Fig. 8). The corresponding temporal trace of signal intensity for the selected area is then calculated by averaging pixel intensities and displayed in the Ca^2+^ trace window. If the Ca^2+^ traces meet the criteria for accepted cells, the manually identified cell can be added to the list of missing cells.

##### Evaluation of CalV2N performance

**Figure 9.**
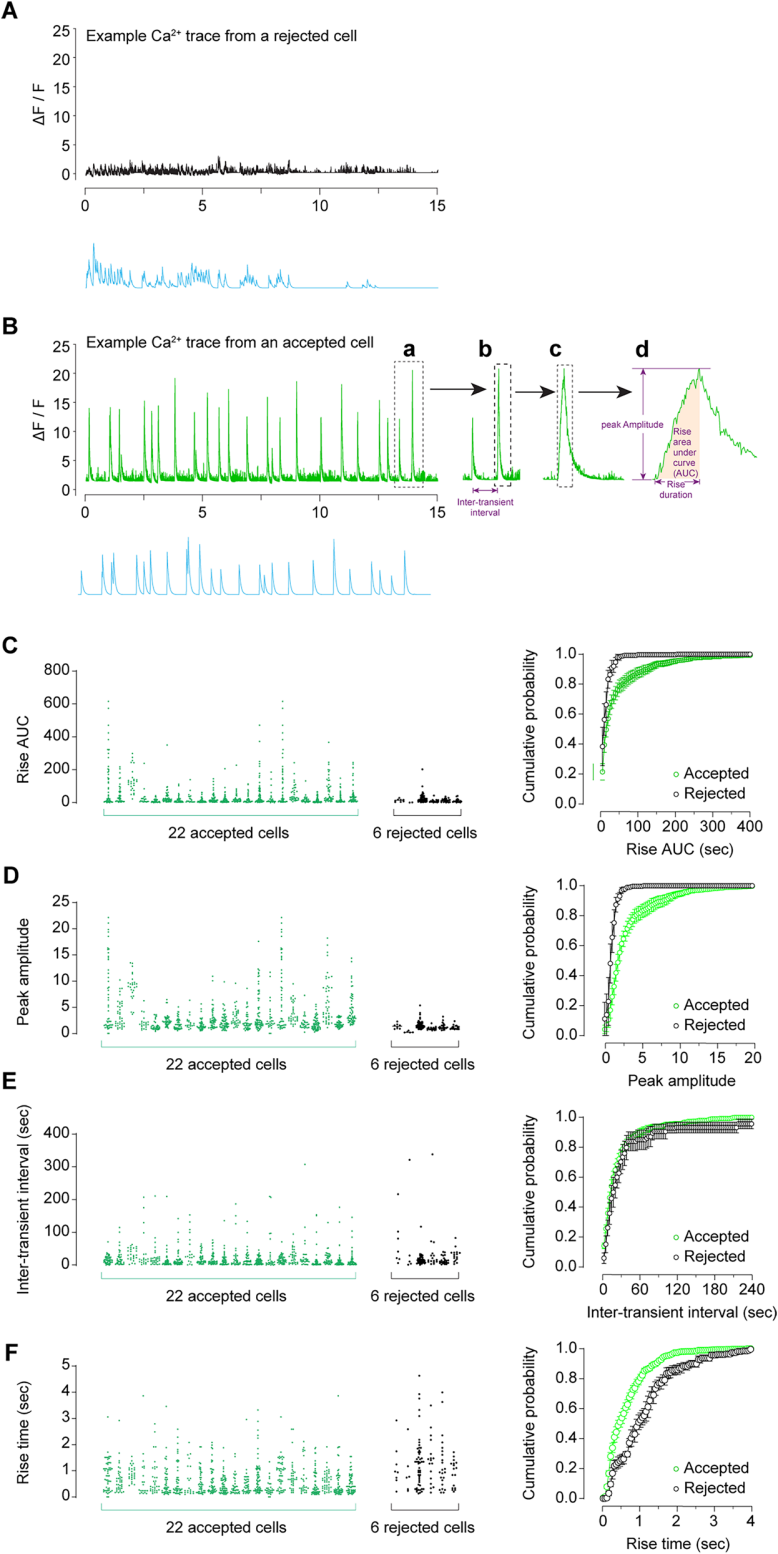
Ca^2+^ transient dynamics in accepted and rejected cells. ***A***, Example Ca^2+^ transient traces from a rejected cell. ***B***, Example Ca^2+^ transient traces from an accepted cell. ***a*** → ***b***, the dash-framed trace in ***a*** was horizontally enlarged to illustrate the measurement of ITI. ***b*** → ***c***, the dash-framed trace in ***b*** was further horizontally enlarged to illustrate the rise and decay of a Ca^2+^ transient. ***c*** → ***d***, the dash-framed trace in **c** was further horizontally enlarged to illustrate the measurements of peak amplitude, the rise AUC, and the rise time. ***C***, The individual rise AUC values of Ca^2+^ transients from 22 accepted cells and 6 rejected cells in M2 randomly selected from a mouse, and the summary data showing the different cumulative probability between accepted versus rejected cells, indicating that accepted cells exhibit more Ca^2+^ transients with larger rise AUC values compared with rejected cells. ***D***, The individual peak amplitude values of Ca^2+^ transients from 22 accepted cells and 6 rejected cells in M2 randomly selected from a mouse, and the summary data showing the different cumulative probability between accepted versus rejected cells, indicating that accepted cells exhibit more Ca^2+^ transients with larger peak amplitude values compared with rejected cells. ***E***, The individual ITI values of Ca^2+^ transients from 22 accepted cells and 6 rejected cells in M2 randomly selected from a mouse, and the summary data showing the similar cumulative probability between accepted versus rejected cells. ***F***, The individual rise time values of Ca^2+^ transients from 22 accepted cells and 6 rejected cells in M2 randomly selected from a mouse, and the summary data showing the different cumulative probability between accepted versus rejected cells, indicating that accepted cells exhibit less Ca^2+^ transients with larger long rise time compared with rejected cells.

Although many CalV2N tools have been developed, there is no evaluation system available to assess the quality of cell identification and Ca^2+^ transient extraction. One of our initial motivations for developing CalTrig was to address this gap by creating a platform to evaluate CalV2N outputs (Fig. 5). Cells identified by CalV2N can be accepted or rejected (Fig. 9*A*,*B*, example traces), and missing cells can be manually added as described above. Further refinement of cell quality evaluation is possible after Ca^2+^ transient detection, using metrics such as rise time, peak amplitude, and inter-transient interval, etc., to review transient kinetics as show in Figure 9*C–F*. If the rate of missing cells is too high (e.g., 10% or higher) and/or the rate of acceptable cells is too low (e.g., 90% or lower), further optimization of CalV2N parameters should be considered. For example, Minian pipeline can be optimized by adjusting the following parameters (Dong et al., 2021).

1. ksize: Controls the denoising step, filtering out electronic noise while preserving important cell details.
2. wnd: Regulates the background removal step, subtracting unwanted light to isolate signals from cells.
3. dl_wnd: Used in the spatial update to focus on nearby cells and adjust the shape of cell signals.
4. sparse_penal: In the spatial update, it controls how detailed or simplified the shapes of the detected cells are. In the temporal update, it balances how much detail is included in the signals from the cells. A higher value makes the signals simpler and less detailed. The best value for this needs to be found through experimentation, as it is hard to estimate in advance.
5. sparsity: Ensures only the most meaningful signals are captured during the temporal update, minimizing noise.

Low SNR may also indicate poor raw data quality, which can be addressed by optimizing AAV preparation (e.g., selecting appropriate subtypes, adjusting titer and volume), improving surgical techniques (slower needle insertion/withdrawal, slower AAV delivery, minimizing bleeding), and enhancing data collection methods (adjusting focal levels, securing MiniScope anchoring, preventing cable twists via sensitive commutator, and ensuring reliable hardware connections).

#### Ca^2+^ transient identification

Transient confirmation can be done in three ways, including manually selecting the start and end points of the transients, autofiltering with specified kinetic parameters or specifying frame number ranges, or running a trained machine learning model.

##### Manual identification

**Figure 10.**
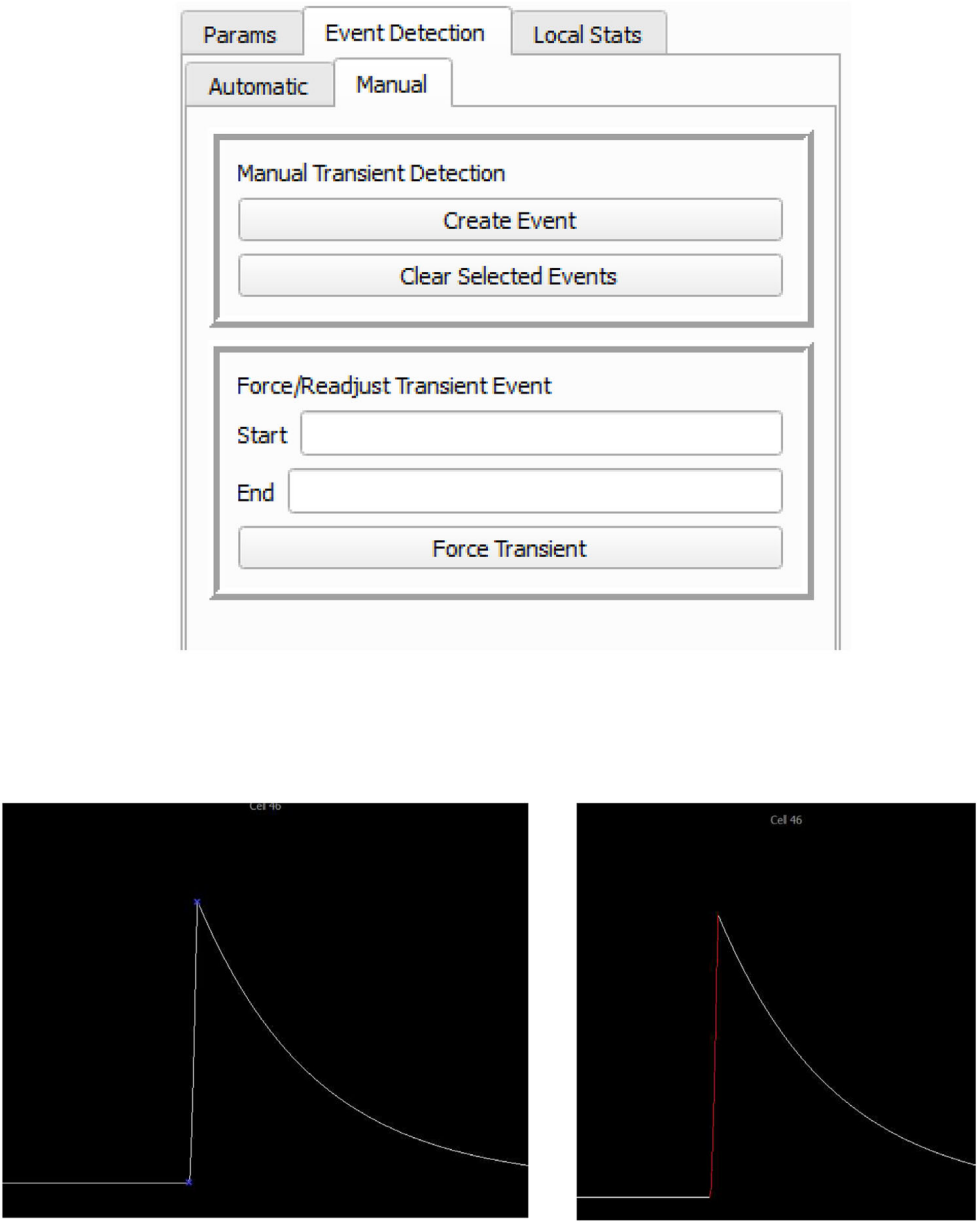
Manual identification of Ca^2+^ transients. Ca^2+^ transients can be directly identified by manually selecting the start and end points of the rising section of Ca^2+^ transients.

Method: Ca^2+^ transients can be directly identified by manually selecting the start and end points of the rising section of Ca^2+^ transients (Fig. 10). Efforts have been made to improve the efficiency of manual identification. For example, the window size of the Ca^2+^ can be preset at a desired size (e.g., 1,000 frames per window width in the *x*-axis, −1 to 20 as the signal range in the *y*-axis), which will allow the visual impression of Ca^2+^ at different time stage, different animals are comparable, assisting a consistent decision-making on Ca^2+^ manual identification. We also set up the shortcut keys to switch the screen window in the Ca^2+^ trace panel. Specifically, clicking A and F allows to jump to the first or the last window, respectively, and clicking S and D allows to jump to the previous or the next screen window, respectively. To account for inaccuracies resulting from human interaction, the selected points would automatically position themselves within a 20-frame window to the local maxima or minima, dependent on their order.

Applications for manual identification: First, directly label the Ca^2+^ transient at the beginning stage of the project when no reference in setting up the parameters for autodetection or no dataset are available to training the machine learning model. It takes ∼5 min to go through a 15 min Ca^2+^ traces. Second, correct, add, or remove the transient spikes after running the parameter-based autodetection to establish the ground truth for training the machine learning model. This would primarily apply to problematic spikes around the specified parameter threshold or those whose rising part is too slow to be confidently identified as a transient event. It takes ∼1 min to go through a 15 min Ca^2+^ traces. Third, to further improve the Ca^2+^ transient identification after processed by an established machine learning model. We expect this reviewing and correction process can be done on 2–4 cells per min. Fourth, when we need to extend the application of a well-trained machine learning model to a different brain region, a different cell type, etc., the machine learning model may provide a compromised predictability in detecting Ca^2+^ transients. Adding a few traces with the manually identified Ca^2+^ transient will set up a new dataset as ground truth for establishing an updated machine learning model to be used in a new task. These four applications of manual identification are illustrated in Figure 7, as indicated by the circled numbers 1–4.

##### Parameter-based automatic identification

Although the variability in Ca^2+^ transient characteristics is well recognized, the fundamental dynamic parameters of Ca^2+^ transients should fall within a certain range. Thus, establishing key dynamic parameters as a digital filter can streamline the Ca^2+^ transient identification process, improving both accuracy and efficiency.

Three Parameters. “Peak Threshold (Δ*F*/*F*)”: First, let us define Δ*F*/*F*, which represents the relative change in fluorescence intensity, where:

- *F*_0_ is the background fluorescence intensity, which is calculated using a moving percentile provided by Caiman (Giovannucci et al., 2019).
- Δ*F* is the difference between the observed fluorescence intensity (*F*) at a given time and the background fluorescence intensity (*F*_0_).

The formula is as follows:

$$\Delta F/F=\frac{F-F_{0}}{F_{0}}.$$

The peak Δ*F*/*F* value within a specified time window is considered as the potential peak of the Ca^2+^ transient. The “Peak Threshold (Δ*F*/*F*)” is the minimum value of peak Δ*F*/*F*. A Ca^2+^ transient is accepted only if its peak Δ*F*/*F* surpasses this threshold.

“Interval Threshold”: This specifies the minimum inter-transient interval (ITI), measured as the frame distance between the initial fames of two adjacent Ca^2+^ transients. If this distance is shorter than the threshold, the transients are merged, with the lower peak combined into the higher peak. However, a peak occurring within the prolonged decay slope of a preceding transient but exceeding the interval threshold is counted as an independent event. Its amplitude is calculated as the difference between its peak Δ*F*/*F* and the Δ*F*/*F* at its rising phase onset.

**Figure 11.**
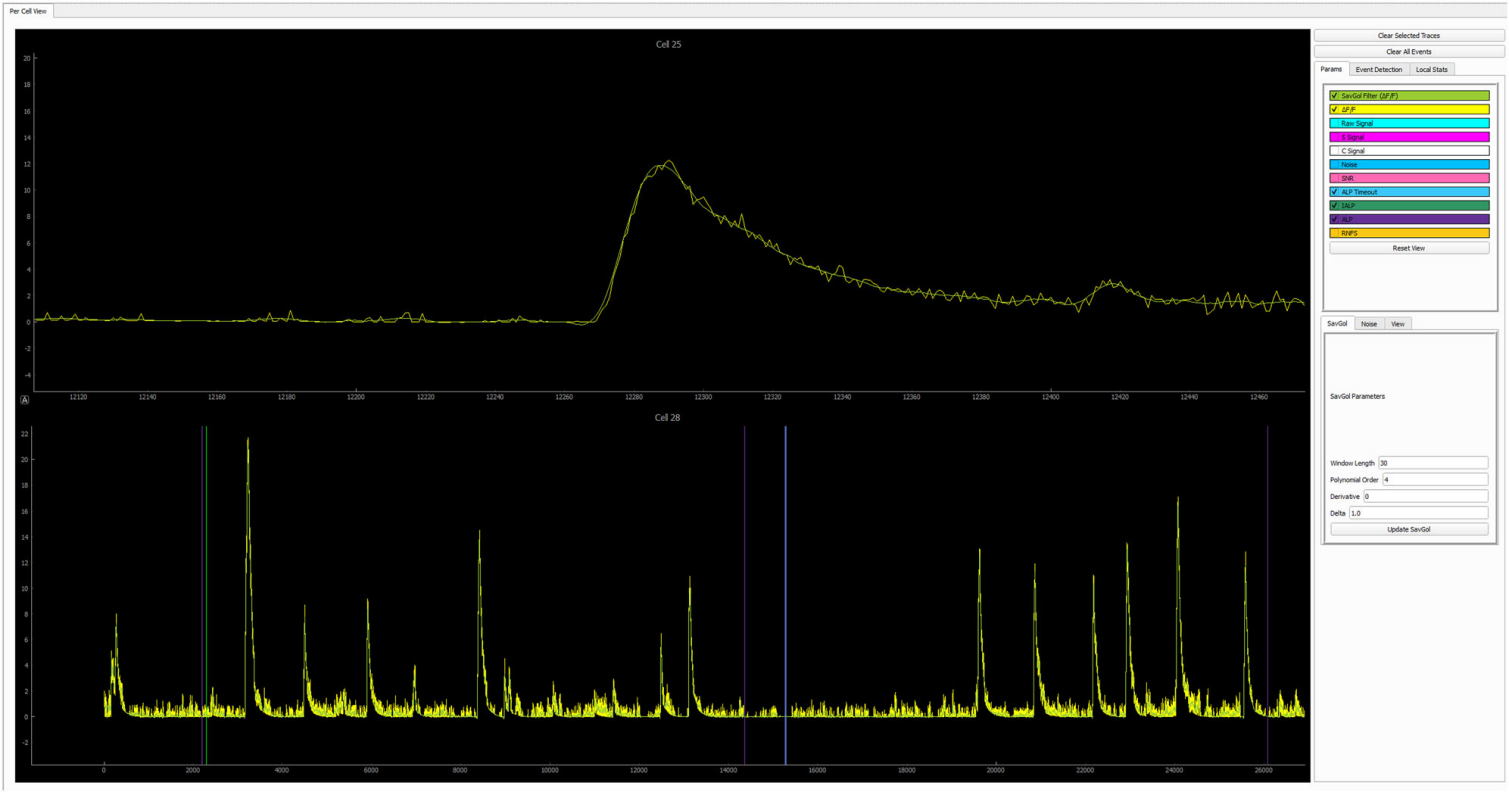
Savitzky–Golay filter. This sets the minimal signal-to-noise ratio (SNR). Using the Savitzky–Golay filter (Steinier et al., 1972; Dai et al., 2017) to smooth Δ*F*/*F* signals, noise is calculated as the difference between the original and filtered Δ*F*/*F*, further smoothed by a rolling window strategy. Then SNR is computed by dividing the smoothed Δ*F*/*F* by the estimated noise.

“SNR Threshold”: This sets the minimal Signal-to-Noise ratio (SNR). Using the Savitzky–Golay filter (Steinier et al., 1972; Dai et al., 2017) to smooth Δ*F*/*F* signals, noise is calculated as the difference between the original and filtered Δ*F*/*F*, further smoothed by a rolling window strategy. Then SNR is computed by dividing the smoothed Δ*F*/*F* by the estimated noise. See more details below and in Figure 11.

##### Signal-to-noise estimation

While verifying transient events, occasional spikes were detected due to background fluctuations rather than specific cellular activity, and in rare cases, noise was misinterpreted as calcium signals. Contemporary methods often use *z*-scores to estimate noise, but this global noise estimation lacks robustness when noise fluctuates over time, potentially leading to the rejection of valid transients during low-noise periods.

To address this concern, we apply the Savitzky–Golay (SavGol) filter (Fig. 11), which smooths noisy data while preserving important features such as peaks and edges (Steinier et al., 1972; Dai et al., 2017). This filter fits successive polynomial functions to adjacent data points, making it ideal for time-series data where preserving peak shape is crucial. In our pilot studies, where we compared the original Δ*F*/*F* with the smoothed Δ*F*/*F* using CalTrig's data exploration tools, the Ca^2+^ transients were well preserved. Therefore, the SavGol filter was chosen as the primary smoothing filter.

Noise estimation for each image frame is calculated by the difference between the original and SavGol-filtered Δ*F*/*F*:

$$\mathrm{Si}g_{\mathrm{noise}}(t)\approx \left|\mathrm{Si}g_{\frac{\Delta F}{F}}-\mathrm{savgol}\left(\mathrm{Si}g_{\frac{\Delta F}{F}}\right)\right|.$$

To further refine the noise estimation, we apply a rolling window function, preventing small value overlaps between the smoothed and nonsmoothed signals. The user can choose smoothing types (average, median, max) and adjust the rolling window size (20 frames is a recommended starting point). SNR is calculated by dividing the smoothed signal by the estimated noise, with a minimum cap (e.g., 0.1) to avoid ineffective or exaggerated values. The resulting SNR effectively mirrors the Δ*F*/*F*, proportionally accentuated or diminished by the noise.

The parameters for the SavGol filter and noise smoothing can be adjusted under the “SavGol” and “Noise” subtabs in the Trace toolbox window. Visualization options for the SavGol-filtered Δ*F*/*F*, noise, and SNR are available under the “Params” subtab for easy analysis in the Ca^2+^ trace window.

At the initial experimental stage, parameter settings are determined using manual identification on a limited number of cells or demo data. As more cells are manually identified, these parameters can be refined to enhance the effectiveness of parameter-based autodetection.

**Figure 12.**
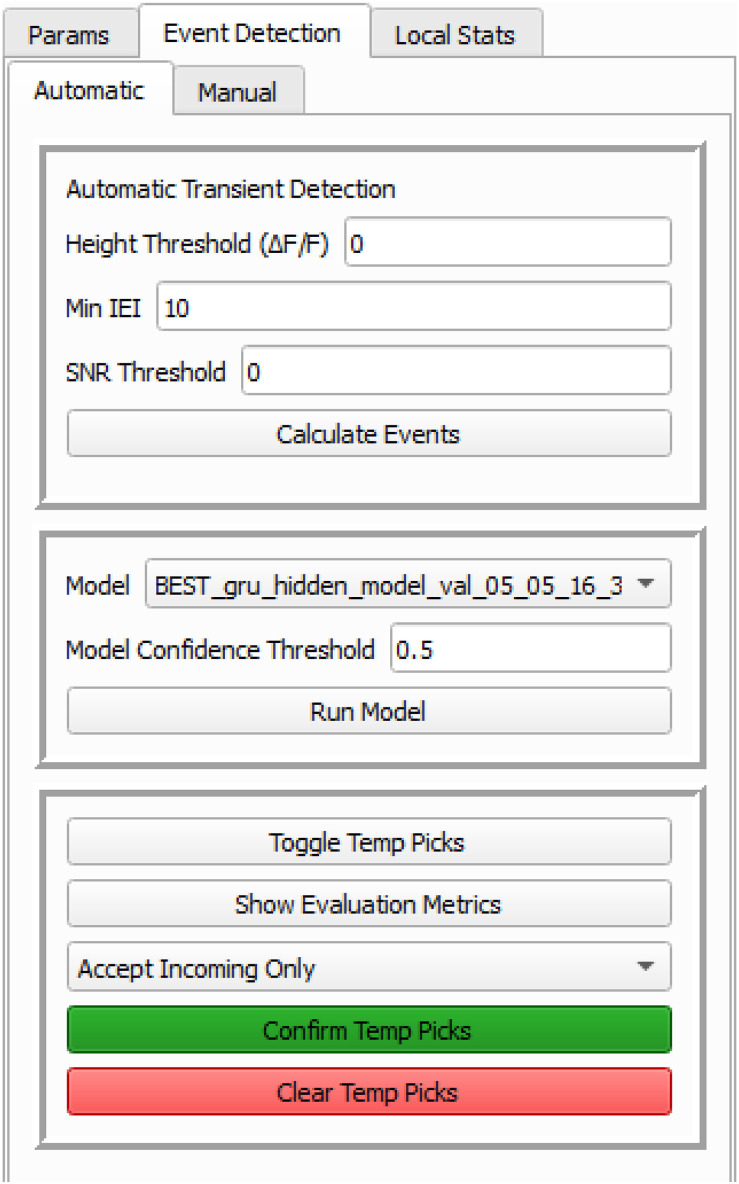
Automatic detection. To enhance detection accuracy, the parameter-based autodetection algorithm starts by including all C peaks in a candidate pool and then filtering down to a valid subset based on three predefined parameters: “Peak Threshold (Δ*F*/*F*),” “Interval Threshold,” and “SNR Threshold.”

Method: Through manual identification, the C signal from CNMF was found to reliably predict Ca^2+^ transients in most cases but prone to false positives. To enhance detection accuracy, the parameter-based autodetection algorithm starts by including all C peaks in a candidate pool, then filtering down to a valid subset based on three predefined parameters: “Peak Threshold (Δ*F*/*F*),” “Interval Threshold,” and “SNR Threshold” (Fig. 12). This is achieved through the following steps.

1. Initial peak detection through local maxima calculation of the C signal.
2. Refine peak selection from left to right by looping the following steps.
3. Remove erroneous peaks if the S signal is zero.
4. Allocate overlapping, continuous S signal to the current peak.
5. Check if the distance to the next peak is shorter than the predefined “Interval Threshold.”
6. If so, merge the current and subsequent peaks, selecting the taller peak.
7. If not, use the S signal to determine the start and end of the transient.
8. Accept the transient selection if in the defined boundaries, the values surpass both the predefined “Peak Threshold (Δ*F*/*F*)” and “SNR Threshold.”

Application: Parameter-based Ca^2+^ transient detection is faster than manual detection, but less predictive than using a well-trained machine learning model. It is particularly feasible after manual identification of a few cells, enabling parameter tuning. Parameter-based detection can then serve as a predetection step to accelerate further manual identification, eventually creating a ground-truth dataset for training machine learning models. This method also helps refine or update machine learning models for different experimental conditions (Figs. 15, 18–20).

##### Detection using machine learning model

*Feature selection for machine learning model*: In selecting the appropriate architecture for the machine learning model, we aimed to incorporate key signals gained from the “ground truth” Ca^2+^ transient verification by manual detection process. We identified two signals, i.e., C and Δ*F*/*F*, as sufficient for decision-making. The determination of whether a given time-step contains a Ca^2+^ transient can be inferred from a 100-frame window centered by the frame of interest. A corresponding dataset, denoted E, was created by marking the initial and peaking frames within the context of a dynamic rise in Ca^2+^ transients.

**Figure 13.**
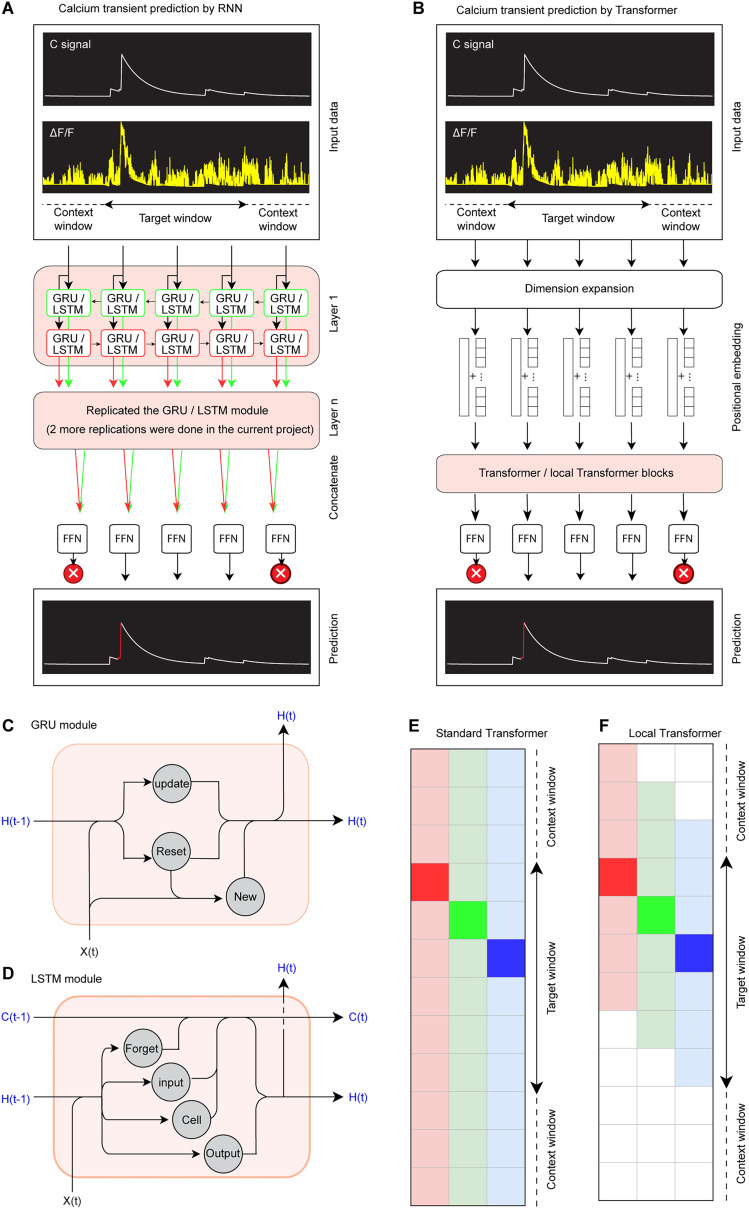
Flowchart demonstrating Ca^2+^ transient validation via machine learning models. ***A***, Application of RNNs in predicting Ca^2+^ transients. The input consists of the C signal and Δ*F*/*F* traces. A sequence of GRUs or LSTM modules processes the temporal dynamics of the Ca^2+^ transient. The network includes multiple stacked layers, with fully connected layers (FNN) at the final stage to produce the predicted Ca^2+^ transient. ***B***, Application of Transformer models for predicting Ca^2+^ transients. The input is first expanded through a dimensional transformation step, followed by repeated attention-based self-mapping in either a standard Transformer or a local Transformer architecture. The processed features are then passed through a feedforward network (FNN) to generate the predicted Ca^2+^ transient. ***C***, ***D***, Diagrams showing the internal operations of two variants of RNN, i.e., GRU module (***C***) and LST module (***D***). The GRU uses two gates: the update gate, which controls the amount of information passed to the next step, and the reset gate, which determines how much of the previous information to forget. These gates modify the hidden state *H*(*t*) at the current time-step based on the input *X*(*t*) and the previous hidden state *H*(*t*−1), leading to a new hidden state *H*(*t*). The LSTM module architecture is depicted, showcasing its cell structure. It uses three gates: forget, input, and output gates to control the flow of information. The input *X*(*t*), the previous cell state *C*(*t*−*1*), and the previous hidden state *H*(*t*−1) are processed through these gates to update the cell state *C*(*t*) and produce the new hidden state *H*(*t*). ***E***, ***F***, Diagram showing the Transform (***E***) and local Transformer (***F***) mechanisms. Multiple columns represent the same set of data. Nontranslucent colored regions highlight the specific values being processed, while translucent colors represent comparison values within the self-attention mechanism. No color denotes values excluded from evaluation, enforcing global attention to all time points. Local Transformer mechanism. Unlike the standard Transformer, the local Transformer focuses only on a subset of adjacent time points, restricting the attention scope to a local temporal window. This reduces computational complexity while maintaining essential local dependencies in Ca^2+^ transient predictions. Data input format: The models receive sequential time-series data of C signals and Δ*F*/*F* traces as input. Data output format: The models predict Ca^2+^ transients by generating the time stamp of the initial and ending frame for each Ca^2+^ transients, which will be saved in ***E***. FNN, Feedforward neural network.

*Selection of machine learning models*: Ca^2+^ transient data are inherently time-series data with a long sequence of many neurons firing at different times, where the signal at any given frame is highly relevant to past and upcoming activity. Thus, Recurrent Neural Networks (RNNs) (Durstewitz et al., 2023) and Transformers (Chandra et al., 2023; Bian et al., 2024) are selected as the candidate machine learning models for Ca^2+^ transient detection due to their ability or potential to handle the temporal dynamics of Ca^2+^ signals in neurons (Fig. 13).

The core of its architecture lies in the RNN cells and its gating mechanisms, which processes inputs sequentially, creating a representation of the data in the context of the preceding timestamps. In the case of Ca^2+^ transient data, two features (i.e., C, Δ*F*/*F*) used for training will be incorporated into the hidden state (*H*) and passed to the subsequent cell (*C*). We tested two RNN variants: Long Short-Term Memory (LSTM; Jiang et al., 2020; Alfattni et al., 2021) and GRU (Wang et al., 2020; Merkelbach et al., 2023). Given the importance of both forward and backward context in detecting Ca^2+^ transients, we employed a bidirectional RNN. The outputs from both directions are concatenated and passed through a feedforward layer to generate the final prediction.

One significant limitation of the RNN is due to the fixed size of its hidden state and the sequential nature of the architecture, causing early time-step bias. To address this, we trained a Transformer, which uses self-attention to compare the relationship between all input values simultaneously, rather than processing sequentially (Choi and Lee, 2023; Liu et al., 2023). This approach allows the model to attend to both low- and high-intensity values that occur at different temporal distances from the Ca^2+^ transient, improving prediction accuracy. Recent advances in Natural Language Processing have introduced the Local Transformer, which limits attention to a subset of nearby inputs (Boffi and Vanden-Eijnden, 2024; Zhao et al., 2024). This model could help reduce errors by focusing on local context and ignoring distant irrelevant signals. However, the self-attention mechanism in the Transformer obscures positional information; hence, we need positional embeddings. The trainable embeddings were used in our Transformer. Additionally, we observed that the small dimensionality of the original input data negatively affected training, likely due to the model's inability to effectively encode positional information in low-dimensional data. To address this, we introduced a preliminary dimension expansion layer, which projects the input into a higher-dimensional space before passing it through the encoding layers.

Both RNN and Transformer architectures were implemented in CalTrig using PyTorch (Lam et al., 2022; Orken et al., 2022). For the Transformer, we used the Transformer Encoder module, and for the Local Transformer, we adapted a modified version of the model (Wang, 2020). Parameters for machine learning model training are listed in Table 3.

**Table 3.** Parameters for machine learning model training

Parameters	GRU and LSTM	Transformer	Local Transformer
Hidden size	30	42	32
Number of layers	3	3	3
Loss function	BCEWithLogitsLoss	BCEWithLogitsLoss	BCEWithLogitsLoss
Optimizer	Adam	Adam	Adam
Initial learning rate	0.001	0.001	0.001
Section Length	200	200	200
Slack	50	50	50
number of Heads		2	1
Local Attention Window Size			10
Look forward/backward Size			5 Local Attention Windows

*Data Preprocessing*: The input data consisted of two signals (i.e., C, Δ*F*/*F*) representing 15 min time intervals of 27,000 frames per cell, captured at a sampling rate of 30 Hz. Each segment was normalized relative to the highest value within the cell, per data type. We tested two data generation and loading approaches:

Discrete Sample Chunking: We defined three variables—sequence length, slack, and rolling parameter. The sequence length specifies the number of frames the model is trained on to make predictions, typically set to ∼200 frames (∼6.6 s) in our testing. Considering the slow dynamics of Ca^2+^ transients, which may last 10–15 s, increasing the segment length could be explored in future optimizations to further improve transient detection. The slack variable determines the extra context length on either side of a given sequence, providing necessary context for making predictions without being used for the predictions themselves. This slack is usually set between 50 and 100 frames. For sequences at the edges of the 27,000-frame segment, zero padding is applied to match the slack length and maintain consistency. Lastly, the rolling parameter defines the windowing approach for generating data, allowing overlapping sequences to be extracted for model training.

Ca^2+^ transient events account for only 2–3% of the overall data. To avoid biasing the model toward detecting non-Ca^2+^ transients, we initially applied class weighting, which resulted in a poor precision score. We determined that it was due to the underemphasizing of noisy data within training, whose signal characteristics were more similar to transient activity rather than an empty signal. This resulted in a model that considered any activity including noise to be a transient event. We opted instead to implement stratification where we ensured that only samples containing ground-truth transient events or positive values from the C array were included. The C array was used as a reference to give the model insight into problematic spikes identified by the CNMF process but deemed invalid by the verifier. During classification, we average all outputs for a single time-step to address overlapping predictions.

**Figure 14.**
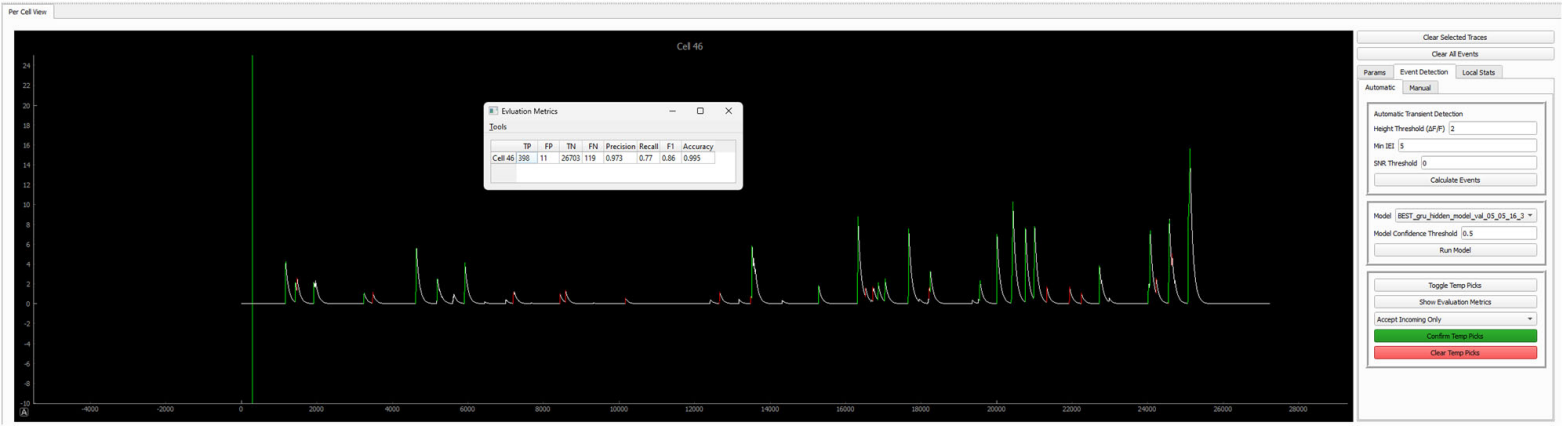
Machine learning evaluation. We used Precision, Recall, F1, and macro F1 as key metrics to evaluate the performance of machine learning model in predicting Ca^2+^ transients versus non-Ca^2+^ transients.

*Key metrics for validation*: We used Precision, Recall, F1, and macro F1 as key metrics to evaluate the performance of machine learning model in predicting Ca^2+^ transients versus non-Ca^2+^ transients (Fig. 14). There are four types of predictions as shown in Tables 4 and 5: true positive (TP) predicting the positive as positive, true negative (TN) predicting the negative as negative, false positive (FP) predicting the negative as positive, and false negative (FN) predicting the positive as negative.

**Table 4.** Event to be predicted: Ca^2+^ transients

	Predicted Ca^2+^ transients	Predicted non-Ca^2+^ transients
Actual Ca^2+^ transients	TP	FN
Actual non-Ca^2+^ transients	FP	TN

**Table 5.** Event to be predicted: non-Ca^2+^ transients

	Predicted Ca^2+^ transients	Predicted non-Ca^2+^ transients
Actual Ca^2+^ transients	TN	FP
Actual non-Ca^2+^ transients	FN	TP

Precision measures how many of the positive predictions (TP + FP) are actually positive (TP). (It focuses on the quality of the positive predictions.)

$$\mathrm{Precision}=\frac{\mathrm{TP}}{\mathrm{TP}+\mathrm{FP}}.$$

Recall measures how many of the actual positive instances (TP + FN) are correctly predicted as positive (TP). (It focuses on the ability to find all positive cases.)

$$\mathrm{Recall}=\frac{\mathrm{TP}}{\mathrm{TP}+\mathrm{NF}}.$$

F1 score is the harmonic mean of Precision and Recall, balancing both metrics.

$$F1=2\times \frac{\mathrm{Precision}\times \mathrm{Recall}}{\mathrm{Precision}+\mathrm{Recall}}.$$

Macro F1 is the average of the F1 scores calculated for each class, i.e., Ca^2+^ transients and non-Ca^2+^ transients.

$$\mathrm{macro}\;\;F1=\;\frac{Ca^{2+}\;\mathrm{transient}\;F1+\mathrm{nonC}a^{2+}\;\mathrm{Transient}\;\;F1}{2}.$$

**Figure 15.**
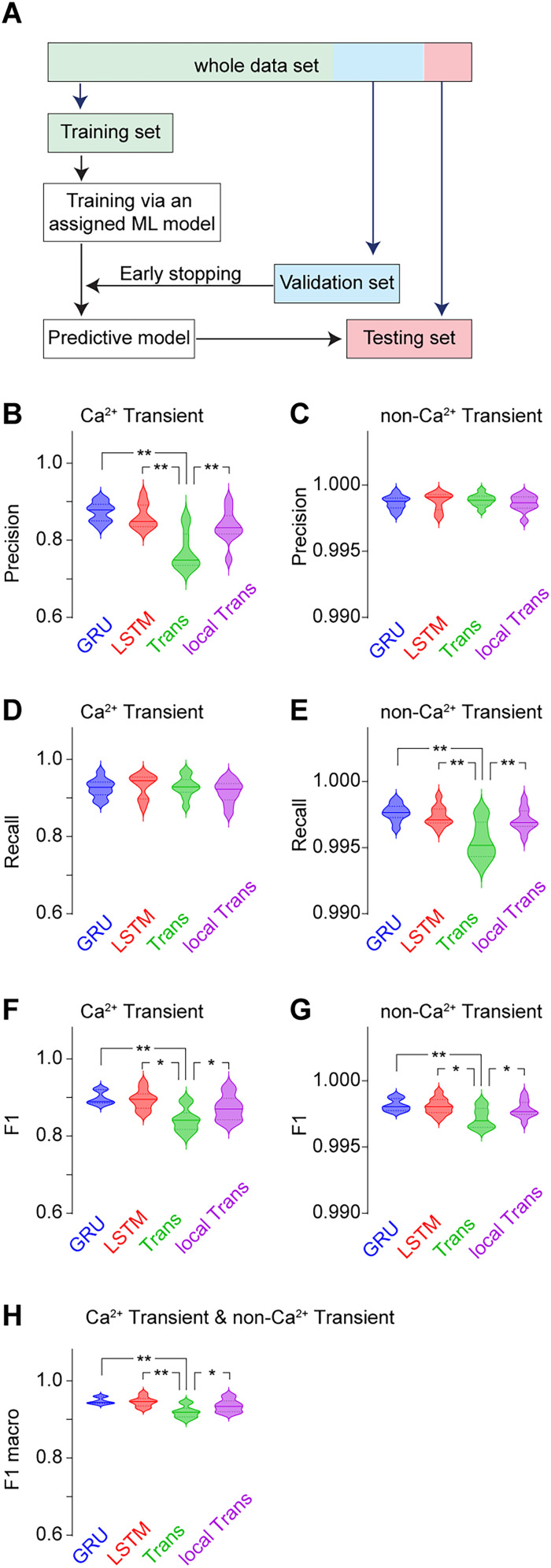
GRU module has the best predictability of Ca^2+^ transients. ***A***, Shared strategy for assigning data to training, validation, and testing across four machine learning models. ***B***, ***C***, Precision varied significantly in Ca^2+^ transient prediction (***B***, *F*_(3,36)_ = 14.2, *p* < 0.01), but remained similar in no Ca^2+^ transient prediction (***C***, *F*_(3,36)_ = 0.4, *p* = 0.78) in four machine learning models. ***D***, ***E***, Recall remained similar in Ca^2+^ transient prediction (***D***, *F*_(3,36)_ = 0.6, *p* = 0.63) but varied in Ca^2+^ transient prediction (***E***, *F*_(3,36)_ = 9.4, *p* < 0.01) in four machine learning models. ***F****,**G***, Significant differences in F1 scores of Ca^2+^ transient prediction (***F***, *F*_(3,36)_ = 9.3, *p* < 0.01) and no Ca^2+^ transient prediction (***G***, *F*_(3,36)_ = 4.8, *p* < 0.01) in four machine learning models. ***H***, Significant differences in macro F1 scores (*F*_(3,36)_ = 9.3, *p* < 0.01) in four machine learning models. Each machine learning model was trained using data from 226 cells from 4 mice, with 28 cells allocated to the validation set and 28 cells to the testing set. Each experimental group consisted of 10 tests. Data were analyzed by one-way ANOVA, followed by Bonferroni post hoc test. *p < 0.05; **p < 0.01.

For evaluation, cells were randomly separated into training (80%), validation (10%), and testing (10%) sets (Fig. 15*A*). Manual identification, parameter-based identification, and machine learning model-based identification can be integrated in detecting Ca^2+^ (Fig. 7).

#### Data export

All the detected Ca^2+^ transient information can be extracted. A few examples are listed below. This will directly aid in statistical analyses and figure preparation for sharing and publishing.

##### Animal-wide data export

**Figure 16.**
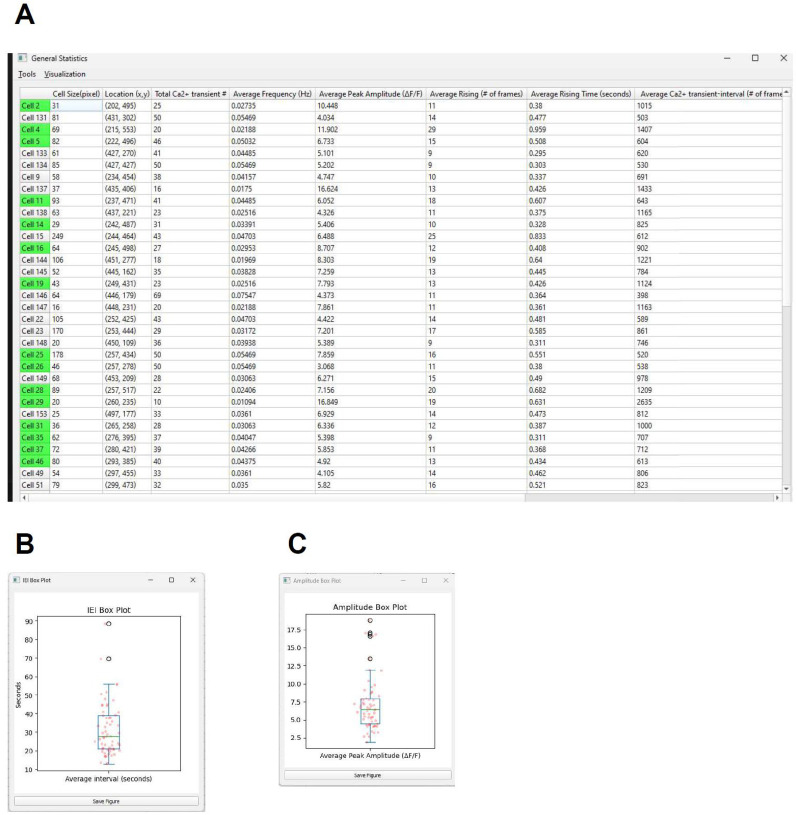
Animal-wide data export. ***A***, A data table is generated, with each row representing one of the CalS2N-detected cells and each column providing specific readout as a general description of that cell. ***B***, ***C***, Average peak amplitude (***B***) and inter-transient interval (ITI, ***C***) can be directly generated and saved as edit able SVG files for publication purposes.

A data table is generated, with each row representing one of the CalV2N-detected cells and each column providing specific readout as a general description of that cell (Fig. 16*A*). From left to right, the columns include the following: Cell ID, Cell Size (number of pixels), Footprint Location (*x*, *y*), Total Ca^2+^ Transient Count, Frequency (Hz), Average Amplitude (Δ*F*/*F*), Average Rising (# of frames), Average Rising Time (seconds), Average Interval (seconds), Standard Deviation (denoted Std, Δ*F*/*F*), Mean Absolute Deviation (denoted MAD, Δ*F*/*F*), Average Peak Amplitude (Δ*F*/*F*), and Category (Verified, Rejected, Missing). The data can be easily copied to the clipboard for further processing in other applications like Excel (Microsoft 365) or Prism (GraphPad). Figures, such as box plots showing the 25, 50, and 75% values for metrics like Average Peak Amplitude or ITI (Fig. 16*B*,*C*), can be directly generated and saved as editable SVG files for publication purposes.

##### Cell-wide data export

**Figure 17.**
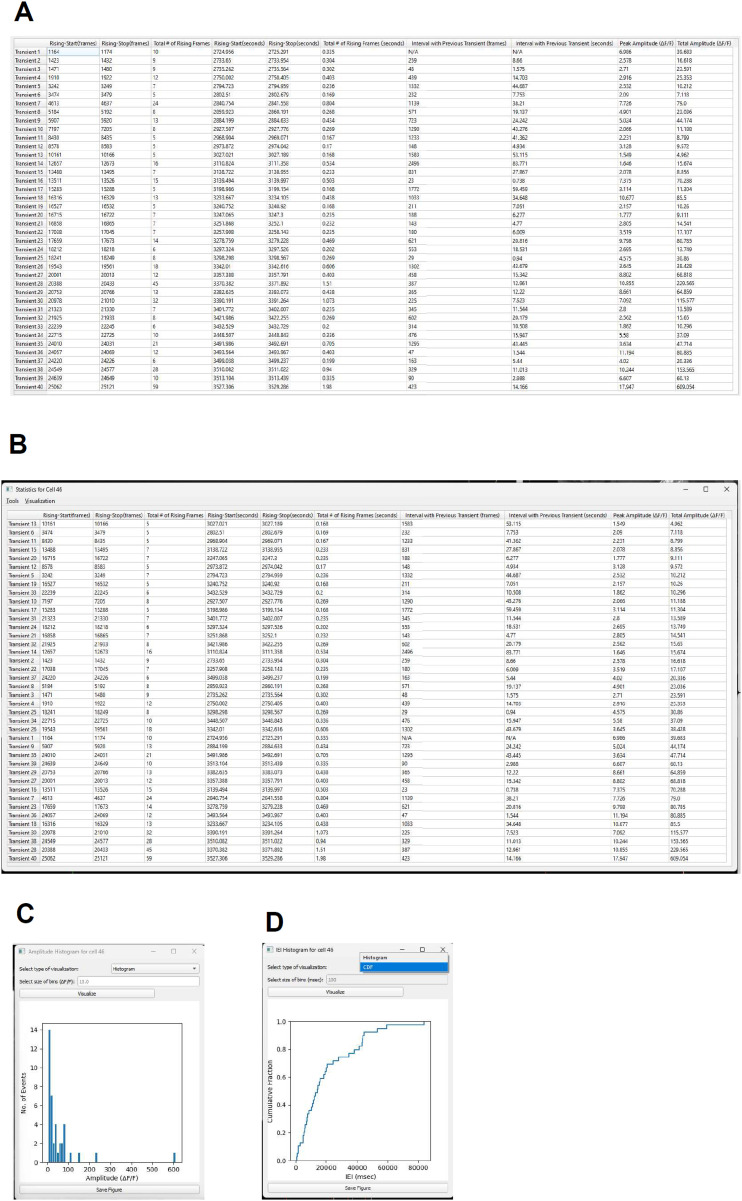
Cell-wide data export. ***A***, A data table is generated for a selected cell, with the top row displaying column titles and the subsequent rows representing individual Ca^2+^ transients detected by CalTrig, one transient per row. ***B***, The column title (e.g., Total Amplitude) can be selected to sort the data based on the information in the selected column. ***C***, ***D***, Figures such as amplitude distribution (***C***) and ITI frequency histograms (***D***) can be created directly from this data and saved as editable SVG files for publication.

A data table is generated for a selected cell, with the top row displaying column titles and the subsequent rows representing individual Ca^2+^ transients detected by CalTrig, one transient per row (Fig. 17*A*). The columns include the following: Rising Start (frames), Rising Stop (frames), Total Rising Frames, Rising Start (seconds), Rising Stop (seconds), Total Rising Time (seconds), Interval with Previous Transient (frames), Peak Amplitude (Δ*F*/*F*), and Total Amplitude (Δ*F*/*F*, calculated as the area under the rising slope). The column title can be selected to sort the data based on the information in the selected column (Fig. 17*B*). This table can be copied to the clipboard for further processing in applications like Excel (Microsoft 365) or Prism (GraphPad). Figures such as Amplitude Distribution or ITI Frequency Histograms (Fig. 17*C*,*D*) can be created directly from this data and saved as editable SVG files for publication.

##### Maximum projection image

An editable image file, including the footprints of detected or verified cells for an animal, can be created for data sharing and publication purposes.

### Statistical analysis

**Figure 18.**
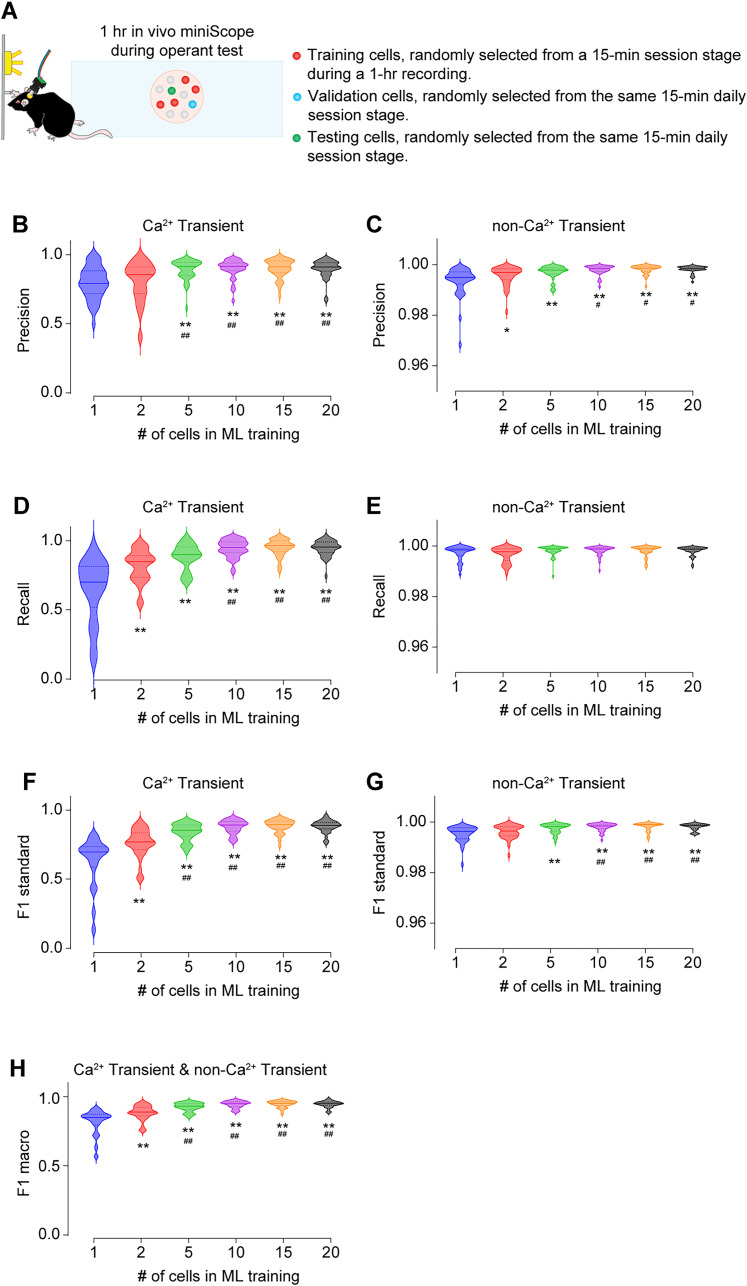
Prediction of Ca^2+^ transients or no Ca^2+^ transients across different numbers of cells in machine learning training. ***A***, Strategy of randomly picking up cells for training, validation, and testing within the same session stage on the same day. ***B****,**C***, Increasing the number of cells in machine learning training model significantly improved the precision in predicting Ca^2+^ transients (***B***, *F*_(5,234)_ = 11.0, *p* < 0.01) and no Ca^2+^ transients (***C***, *F*_(5,234)_ = 12.8, *p* < 0.01). ***D***, ***E***, Increasing the number of cells in machine learning training model significantly improved the recall in predicting Ca^2+^ transients (***D***, *F*_(5,234)_ = 40.9, *p* < 0.01) and no Ca^2+^ transients (***E***, *F*_(5,234)_ = 2.8, *p* = 0.02). ***F****,**G***, Increasing the number of cells in machine learning training model significantly improved the F1 scores in predicting Ca^2+^ transients (***F***, *F*_(5,234)_ = 46.7, *p* < 0.01) and no Ca^2+^ transients (***G***, *F*_(5,234)_ = 12.0, *p* < 0.01). ***H***, Increasing the number of cells in machine learning training model significantly improved the macro F1 scores in predicting Ca^2+^ transients and no Ca^2+^ transients (***H***, *F*_(5,234)_ = 46.6, *p* < 0.01). Data were obtained using the GRU model. Each experimental group consisted of 10 tests per mouse, with 4 mice per group, resulting in a total of 40 data points per experimental group. Data were analyzed by one-way ANOVA, followed by Bonferroni post hoc test. *p < 0.05; **p < 0.01, compared with the machine learning model trained by 1 cell. ^#^*p* < 0.05; ^##^0.01, compared with the machine learning model trained by 2 cells. 40 testing cells in each group.

**Figure 19.**
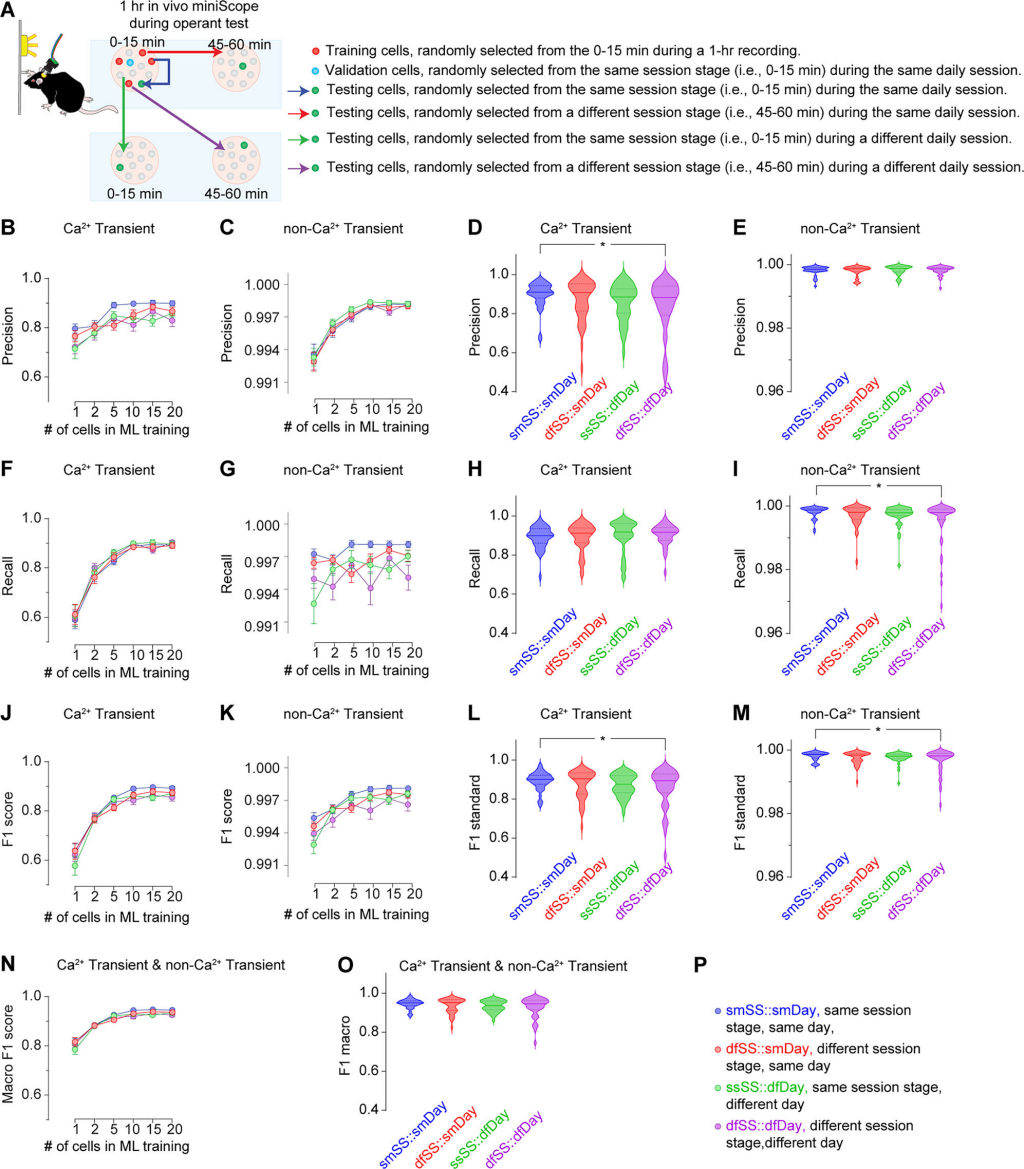
Prediction of Ca^2+^ transients or no Ca^2+^ transients within the same mice. ***A***, Four strategies for sourcing cells within the M2 area from a mouse for machine learning model training, validation, and testing. ***B–E***, The training cell number, and the testing cell source, but not their interactions, significantly affected the Precision in predicting Ca^2+^ transients (***B***, training cell # *F*_(5,936)_ = 21.4, *p* < 0.01; testing cell source *F*_(3,936)_ = 10.0, *p* < 0.01; training cell number × testing cell source interaction *F*_(15,936)_ = 0.8, *p* = 0.64). The training cell number, but not the testing cell source or their interactions, affected the Precision in predicting no Ca^2+^ transients (***C***, training cell number *F*_(5,936)_ = 71.7, *p* < 0.01; testing cell source *F*_(3,936)_ = 1.1, *p* = 0.36; training cell number × testing cell source interaction *F*_(15,936)_ = 0.3, *p* = 0.99). When 20 cells were included in the machine learning training model, the Precision in predicting Ca^2+^ transients (***D***, *F*_(3,156)_ = 2.7, *p* = 0.046), but not in predicting no Ca^2+^ transients (***E***, *F*_(3,156)_ = 0.2, *p* = 0.93), in testing cells from different session stage on different day reduced. ***F–I***, The training cell number, but not the testing cell source or their interactions, significantly affected the Recall in predicting Ca^2+^ transients (***F***, training cell number *F*_(5,936)_ = 126.0, *p* < 0.01; testing cell source *F*_(3,936)_ = 0.8, *p* = 0.49; training cell number × testing cell source interaction *F*_(15,936)_ = 0.3, *p* = 0.99). The number of cells, the testing cell source, but not their interactions, significantly affected the Recall in predicting no Ca^2+^ transients (***G***, training cell number *F*_(5,936)_ = 2.7, *p* = 0.02; testing cell source *F*_(3,936)_ = 11.4, *p* < 0.01; training cell number × testing cell source interaction *F*_(15,936)_ = 1.5, *p* = 0.10). When 20 cells were included in the machine learning training model, the Recall in predicting no Ca^2+^ transients (***I***, *F*_(3,156)_ = 3.3, *p* = 0.02), but not in predicting the Ca^2+^ transients (***H***, *F*_(3,156)_ = 0.4, *p* = 0.78), in testing cells from different session stage on different day reduced. ***J–M***, The training cell number and the testing cell source, but not their interactions, significantly affected the F1 scores in predicting Ca^2+^ transients (***J***, training cell number *F*_(5,936)_ = 130.0, *p* < 0.01; testing cell source *F*_(3,936)_ = 3.0, *p* = 0.03; training cell number × testing cell source interaction *F*_(15,936)_ = 0.7, *p* = 0.74). Similarly, the number of cells and the testing cell source, but not their interactions, significantly affected the F1 scores in predicting no Ca^2+^ transients (***K***, training cell number *F*_(5,936)_ = 32.3, *p* < 0.01; testing cell source *F*_(3,936)_ = 8.8, *p* < 0.01; training cell number × testing cell source interaction *F*_(15,936)_ = 1.2, *p* = 0.28). When 20 cells were included in the machine learning training model, the Recall in predicting Ca^2+^ transients (***L***, *F*_(3,156)_ = 2.7, *p* = 0.048) and no Ca^2+^ transients (***M***, *F*_(3,156)_ = 2.8, *p* = 0.040) in testing cells from different session stage on different day reduced. ***N***, ***O***, The training cell number, and the testing cell source, but not their interactions, significantly affected the macro F1 scores in predicting Ca^2+^ transients and no Ca^2+^ transients (***N***, training cell number *F*_(5,936)_ = 128.5, *p* < 0.01; testing cell source *F*_(3,936)_ = 3.1, *p* = 0.03; training cell number × testing cell source interaction *F*_(15,936)_ = 0.8, *p* = 0.73). When 20 cells were included in the machine learning training model, the macro F1 scores in predicting Ca^2+^ transients and no Ca^2+^ transients (***O***, *F*_(3,156)_ = 2.8, *p* = 0.041) in testing cells from different session stage on different day reduced. ***P***, Legends showing the color-coded abbreviations for four experimental groups. Data were obtained using the GRU model. Each experimental group consisted of 10 tests per mouse, with 4 mice per group, resulting in a total of 40 data points per experimental group. Data were analyzed by two-way ANOVA (***B***,***C***,***F***,***G***,***J***,***K***,***N***) or one-way ANOVA (***D***,***E***,***H***,***I***,***L***,***M***,***O***), followed by Bonferroni post hoc test. *p < 0.05; **p < 0.01. 40 testing cells in each group.

**Figure 20.**
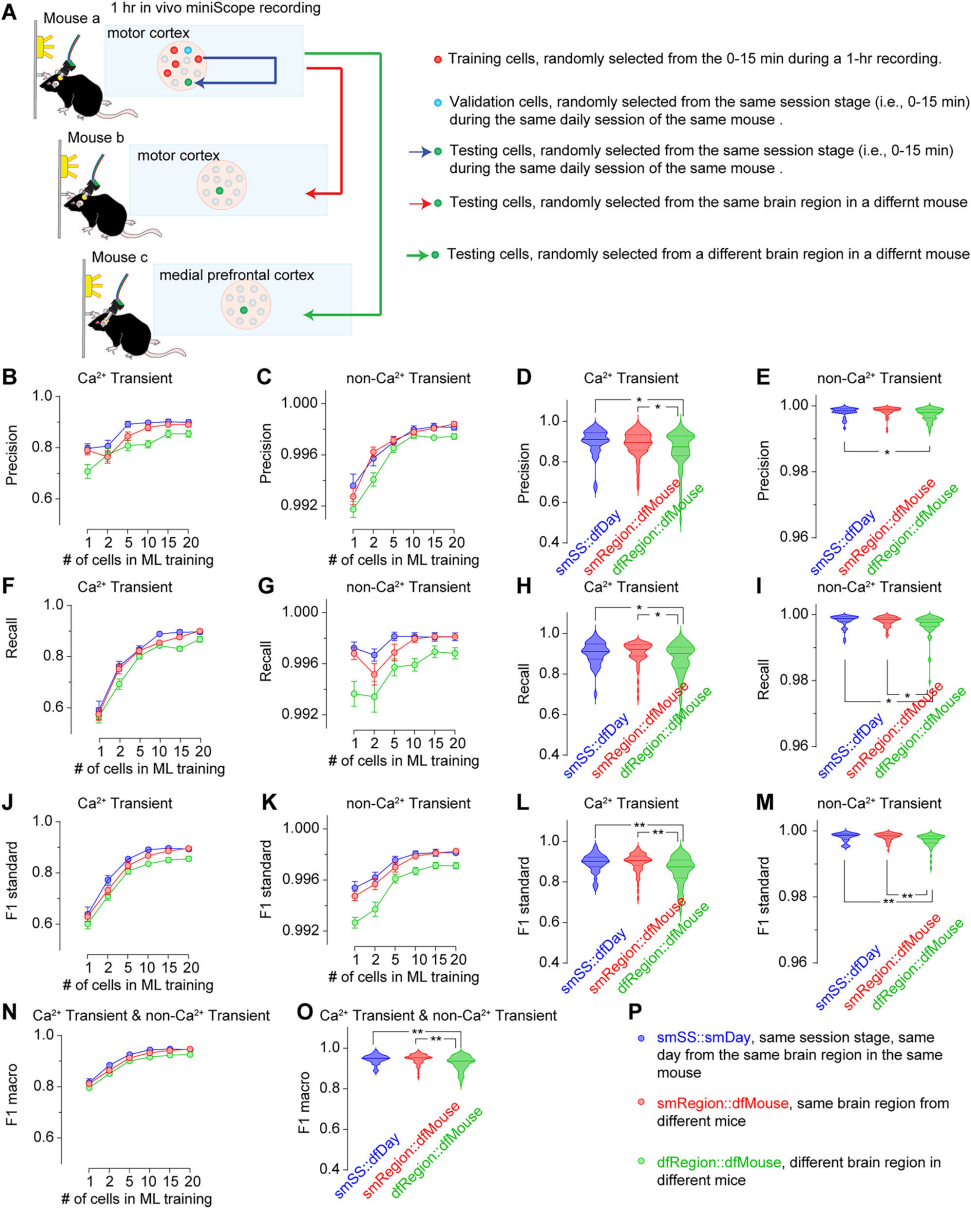
Prediction of Ca^2+^ transients or no Ca^2+^ transients in different mice. ***A***, Three strategies for sourcing testing cells (smSS::smDay, between mice, different regions, see more details in panel ***P***). ***B–E***, The training cell number, and the testing cell source, but not their interactions, significantly affected the Precision in predicting Ca^2+^ transients (***B***, training cell number *F*_(5,942)_ = 24.4, *p* < 0.01; testing cell source *F*_(2,942)_ = 19.2, *p* < 0.01; training cell number × testing cell source interaction *F*_(10,942)_ = 1.1, *p* = 0.37) and the no Ca^2+^ transients (***C***, training cell number *F*_(5,942)_ = 74.1, *p* < 0.01; testing cell source *F*_(2,942)_ = 11.6, *p* < 0.01; training cell number × testing cell source interaction *F*_(10,942)_ = 1.0, *p* = 0.43). When 20 cells were included in the machine learning training model, the Precision in predicting Ca^2+^ transients (***D***, *F*_(2,157)_ = 4.7, *p* = 0.01) and no Ca^2+^ transients (***E***, *F*_(2,157)_ = 6.2, *p* < 0.001) in testing cells from different regions in different mice reduced. ***F–I***, The training cell number, but not the testing cell source or their interactions, significantly affected the Recall in predicting Ca^2+^ transients (***F***, training cell number *F*_(5,942)_ = 145.6, *p* < 0.01; testing cell source *F*_(2,942)_ = 3.7, *p* = 0.02; training cell number × testing cell source interaction *F*_(10,942)_ = 0.7, *p* = 0.68). The training cell number and the testing cell source, but not their interactions, significantly affected the Recall in predicting no Ca^2+^ transients (***G***, training cell number *F*_(5,942)_ = 8.7, *p* < 0.01; testing cell source *F*_(2,942)_ = 22.9, *p* < 0.01; training cell number × testing cell source interaction *F*_(10,942)_ = 0.9, *p* = 0.58). When 20 cells were included in the machine learning training model, the Recall in predicting Ca^2+^ transients (***H***, *F*_(2,157)_ = 4.4, *p* = 0.01) and no Ca^2+^ transients (***I***, *F*_(2,157)_ = 5.3, *p* < 0.01) in testing cells from different regions in different mice reduced. ***J–M***, The training cell number, and the testing cell source, but not their interactions, significantly affected the F1 scores in predicting Ca^2+^ transients (***J***, training cell number *F*_(5,942)_ = 165.7, *p* < 0.01; testing cell source *F*_(2,942)_ = 17.6, *p* < 0.01; training cell number × testing cell source interaction *F*_(10,942)_ = 0.3, *p* = 0.98) and the no Ca^2+^ transients (***K***, training cell number *F*_(5,942)_ = 55.8, *p* < 0.01; testing cell source *F*_(2,942)_ = 40.4, *p* < 0.01; training cell number × testing cell source interaction *F*_(10,942)_ = 1.3, *p* = 0.23). When 20 cells were included in the machine learning training model, the F1 scores in predicting Ca^2+^ transients (***L***, *F*_(2,157)_ = 8.9, *p* < 0.01) and no Ca^2+^ transients (***M***, *F*_(2,157)_ = 9.3, *p* < 0.01) in testing cells from different regions in different mice reduced. ***N***, ***O***, The training cell number, and the testing cell source, but not their interactions, significantly affected the macro F1 scores in predicting Ca^2+^ transients and no Ca^2+^ transients (***N***, training cell number *F*_(5,942)_ = 164.6, *p* < 0.01; testing cell source *F*_(2,942)_ = 18.3, *p* < 0.01; training cell number × testing cell source interaction *F*_(10,942)_ = 0.3, *p* = 0.98). When 20 cells were included in the machine learning training model, the macro F1 scores in predicting Ca^2+^ transients and no Ca^2+^ transients (***O***, *F*_(2,157)_ = 9.0, *p* < 0.01) in testing cells from different regions in different mice reduced. ***P***, Legends showing the color-coded abbreviations for four experimental groups. Data were obtained using the GRU model. Each experimental group consisted of 10 tests per mouse, with 4 mice for smSS::smDay, and 6 mice for smRegion::dfMouse and dfRegion::dfMouse, resulting in a total of 40 data points for smSS::smDay and 60 data points for smRegion::dfMouse and dfRegion::dfMouse. Data were analyzed by two-way ANOVA (***B***,***C***,***F***,***G***,***J***,***K***,***N***) or one-way ANOVA (***D***,***E***,***H***,***I***,***L***,***M***,***O***), followed by Bonferroni post hoc test. *p < 0.05; **p < 0.01. 40 testing cells in each group.

Data were collected from 10 mice in vivo, shown as mean ± SEM in curve graphs (Figs. 19*B*,*C*,*F*,*G*,*J*,*K*,*N*, 20*B*,*C*,*F*,*G*,*J*,*K*,*N*) or the quartile in violin plots (Figs. 15, 18, 19*D*,*E*,*H*,*I*,*L*,*M*,*O*, 20*D*,*E*,*H*,*I*,*L*,*M*,*O*). Using GraphPad Prism 10, statistical significance was assessed by one-way ANOVA or two-way ANOVA, followed by Bonferroni post hoc tests. Statistical significance was considered to be achieved if *p* < 0.05. All raw data for TN, TP, FN, and FP are available in an Excel file, which can be downloaded via the following link: https://github.com/cci-MaLab/Calcium-Transient-Analysis/raw/refs/heads/main/ml_training/caltrig_results/TN%20TP%20FN%20FP%20Raw%20data.xlsx

### Code/software

CalTrig is available in our lab Github station (https://github.com/cci-MaLab/Calcium-Transient-Analysis).

## Results

### Comparison of the four machine learning models

Our goal was to find an efficient machine learning model with high predictability in detecting the Ca^2+^ transients while minimizing computational demands. To achieve this, we compared the performance of two RNN variants, GRU and LSTM, alongside two Transformer models, the standard Transformer and local Transformer. We used a dataset of 226 cells with manually confirmed Ca^2+^ transient as “ground” truth (see more details in Materials and Methods; Fig. 15*A*). Our data showed that the macro F1 score for predicting Ca^2+^ transients and no Ca^2+^ transients was higher with RNN (GRU, 0.948 ± 0.002; LSTM, 0.946 ± 0.004; mean ± SEM, same below) compared with the standard Transformer (0.919 ± 0.005), which was partially improved by local Transformer (0.935 ± 0.005; Fig. 15*H*).

Specifically, for Ca^2+^ transient prediction, the F1 score was higher with RNN (GRU, 0.900 ± 0.005; LSTM, 0.894 ± 0.008) compared with the standard Transformer (0.841 ± 0.010), which was partially improved by local Transformer (0.873 ± 0.010; Fig. 15*F*). In terms of Precision, both GRU (0.873 ± 0.008) and LSTM (0.860 ± 0.010) models outperformed the standard Transformer (0.771 ± 0.015) and the local Transformer (0.835 ± 0.013; Fig. 15*B*). However, the Recall scores across four machine learning models were similar (GRU: 0.926 ± 0.008; LSTM: 0.931 ± 0.010; standard Transformer: 0.929 ± 0.009; local Transformer: 0.916 ± 0.010; Fig. 15*D*), indicating that the F1 score differences were mainly driven by Precision.

For predicting non-Ca^2+^ transients, the precision, recall, and F1 scores were all near perfect (>0.990), due to the consistent nature of non-Ca^2+^ transient signals. When analyzing F1 scores specifically for non-Ca^2+^ transient prediction, similar trends as the macro F1 scores were observed across the four machine learning models (Fig. 15*G*), with differences primarily driven by Recall (Fig. 15*E*) rather than Precision (Fig. 15*C*).

Compared with Transformer models, RNN models exhibited higher Precision in detecting Ca^2+^ transients and better Recall for non-Ca^2+^ transients, both attributable to RNN's ability to minimize misclassification of the non-Ca^2+^ transient signal as Ca^2+^ transient signals. The consistent good Recall for Ca^2+^ transients and Precision values for non-Ca^2+^ transients across all machine learning models indicates that they have a low likelihood of misclassifying Ca^2+^ transients as non-Ca^2+^ transients. The primary challenge in identifying Ca^2+^ transients lies in reducing errors where non-Ca^2+^ transients are mistakenly classified as transients. RNN models outperformed Transformer in addressing this issue, which was partially improved when using the local Transformer. Given its lower variability and faster processing time, GRU is recommended over LSTM. Therefore, all subsequent analyses were conducted using the GRU model.

### Optimization of the cell number of the training dataset

To determine the optimal number of cells to be used as the training dataset, we tested the predictability of GRU model trained by varying numbers of cells, ranging from 1 to 20. When the testing datasets were randomly sampled from the same session stage (i.e., the first 15 min or the last 15 min) in the same 1 h daily session on either Day 1 or Day 5, we observed a significant improvement in the macro F1 score when 5–20 cells were sampled, compared with GRU models trained with only 1 or 2 cells. However, no differences were observed among models trained with 5–20 cells (macro F1 score: 1 cell, 0.817 ± 0.014; 2 cells, 0.885 ± 0.008; 5 cells, 0.926 ± 0.005; 10 cells, 0.945 ± 0.004; 15 cells, 0.947 ± 0.004; 20 cells, 0.946 ± 0.004; Fig. 18*H*). This cell number-dependent predictability is primarily attributable to the model's predictability of the Ca^2+^ transient indicated by its Precision (Fig. 18*B*), Recall (Fig. 18*D*), and F1 scores (Fig. 18*F*) for Ca^2+^ transients, as the Precision (Fig. 18*C*), Recall (Fig. 18*E*), and F1 scores (Fig. 18*G*) for non-Ca^2+^ transient were all near perfect regardless of the cell number of the training dataset. We further explored the effects of cell number when testing cells were randomly sampled from the different session stages within the same 1 h daily session, the same session stage on a different daily session, and the different sessions stage on a different daily session of the same mouse (Fig. 19*A*). In all of these three cases, we found significant improvements of both Ca^2+^ transient prediction (Fig. 19*B*,*F*,*J*,*N*) and the non-Ca^2+^ transient predictions, though the latter were near perfect in most instances (Fig. 19*C*,*G*,*K*,*O*), when five or more cells were sampled for training, compared with the models trained with only 1 or 2 cells. Finally, we extended further by randomly sampling the training datasets from different mice (Fig. 20*A*). We found significant improvements of Ca^2+^ transient prediction (Fig. 20*B*,*F*,*J*,*N*) and the non-Ca^2+^ transient prediction, though the latter was near perfect in most instances (Fig. 20*C*,*G*,*K*,*O*), when using five or more cells, regardless of whether the testing cells were from the same or different brain regions in mice distinct from those used for training. In conclusion, the number of cells used for training is crucial for accurately predicting Ca^2+^ transients. A minimum of 10 cells appears to be sufficient for achieving macro F1 scores above 0.900 when training the GRU model.

### Sharing the machine learning model for predicting testing cells across different time windows in the same mouse

To assess how well a GRU model trained on data from a section of a 1 h daily session can predict Ca^2+^ transients from any other recordings in the same mouse, we randomly sampled testing cells under four conditions: (1) the same session stage in the same daily session (smSS::smDay), (2) a different session stage in the same daily session (dfSS::smDay), (3) the same session stage in a different daily session (smSS::dfDay), and (4) a different session stage in a different daily session (dfSS::dfDay; Fig. 19*A*). When 20 cells were sampled, the macro F1 scores were 0.946 ± 0.004 for smSS::smDay, 0.936 ± 0.006 for dfSS::smDay, 0.934 ± 0.005 for smSS::dfDay, and 0.927 ± 0.008 for dfSS::dfDay (Fig. 19*N*). Thus overall, the predictability remained high when testing cells were sampled from the same mouse used for GRU model training, though the macro F1 score for predicting both Ca^2+^ transients and non-Ca^2+^ transients was affected statistically, but mildly, by the source of testing cells (Fig. 19*N*,*O*).

Specifically, for predicting Ca^2+^ transients, the source of testing cells affected the F1 scores (Fig. 19*J*,*L*), particularly the Precision (Fig. 19*B*,*D*) rather than the Recall (Fig. 19*F*,*H*). When smSS::smDay was set as the gold standard with 20 cells as the benchmark, similar predictability was observed when testing cells were sampled from dfSS::smDay or smSS::dfDay. However, lower Precision and F1 score, but comparable Recall, were noted when testing cells were sampled from dfSS::dfDay.

For predicting non-Ca^2+^ transients, the source also affected the F1 scores (Fig. 19*K*,*M*), primarily impacting the Recall (Fig. 19*G*,*I*), while the Precision remained largely unaffected (Fig. 19*C*,*E*). Similarly, as what was observed for predicting Ca^2+^ transients, we found the non-Ca^2+^ transient prediction was comparable when testing cells were sampled from smSS::smDay, dfSS::smDay, or smSS::dfDay. Lower Recall and F1 score, but similar Precision, for non-Ca^2+^ transient prediction was detected when testing cells were sampled from dfSS::dfDay.

In conclusion, the GRU model can effectively predict Ca^2+^ transients in cells from the same mouse used for training. However, the risk of misclassifying non-Ca^2+^ transients as Ca^2+^ transients increases, though within a narrow range, when testing cells are sampled from a different session stage and a different day.

### Sharing the machine learning model for predicting testing cells from different mice

To evaluate how the GRU model can predict the Ca^2+^ transients from a different mouse, either sharing the same brain region or switching to a different brain region, we used the smSS::smDay sampling strategy as the benchmark and added two more strategies in sampling testing cells: (1) the same brain region in a different mouse (denoted smRegion::dfMouse) and (2) a different brain region in a different mouse (denoted dfRegion::dfMouse). When 20 cells were sampled, the macro F1 scores were 0.946 ± 0.004 for smDay::smSS, 0.947 ± 0.003 for smRegion::dfMouse and 0.927 ± 0.005 dfRegion::dfMouse. Thus, regardless of the brain region specificity, overall predictability remained high when testing cells were sampled from mice different from the one used for GRU training, although the macro F1 score for predicting both Ca^2+^ and non-Ca^2+^ transients was statistically reduced when the testing cells were sampled in dfRegion::dfMouse (Fig. 20*N*,*O*).

Specifically, for predicting Ca^2+^ transients in different mice, the source of testing cells affected the F1 scores (Fig. 20*J*,*L*) by impacting both the Precision (Fig. 20*B*,*D*) and the Recall (Fig. 20*F*,*H*). Lower Precision, Recall, and F1 score were noted when testing cells were sampled from dfRegion::dfMouse, relative to either smSS::smDay or dfRegion::dfMouse.

For predicting non-Ca^2+^ transients, the source also affected the F1 scores (Fig. 20*K*,*M*), by impacting both the Precision (Fig. 19*C*,*E*) and the Recall (Fig. 19*G*,*I*). Lower, although still near perfect, Precision, Recall, and F1 score were noted when testing cells were sampled from dfRegion::dfMouse, relative to either smSS::smDay or dfRegion::dfMouse.

In conclusion, the GRU model can identify Ca^2+^ transients in cells from different mice with a macro F1 beyond 0.900. However, the risk of misclassifying non-Ca^2+^ transients as Ca^2+^ transients or Ca^2+^ transients as non-Ca^2+^ transients increases, though within a narrow range, when testing cells are sampled from a different brain region of a different mouse. There appears to be no significant impact on the F1 score when the testing cells are from the same region of a different mouse.

## Discussion

### Selection of machine learning model

In recent years, several studies using different machine learning methods have been developed utilizing some combination of the CNN, attention-based architectures for cell identification, and subsequent cell extraction (Denis et al., 2020; Bao et al., 2021; Sita et al., 2022). However, they require a significant amount of labeled data as ground truth to achieve adequate predictive performance. This raise concerns in both time investment to train a single model as well as, due to the black box nature of these models, their ability to maintain performance given a substantial change in the input data (such as changes to different experimental animals, another brain region of interest, a different type of neuron).

In this study, we evaluated four machine learning models (GRU, LSTM, Transformer, and Local Transformer) for Ca^2+^ transient detection. Both predictability performance (i.e., precision, recall, and F1 scores) and time efficiency should be considered. First, GRU model is the most time efficient, with a computation time of 287.1 s/epoch, slightly faster than LSTM (290.59 s/epoch) due to its simpler architecture and fewer parameters, making it computationally less expensive. GRU also exhibits strong overall performance in terms of precision, recall, and F1 scores, making it an excellent choice when considering both predictability and computation time. Second, LSTM takes marginally longer than GRU but performs slightly worse across multiple predictability metrics for Ca^2+^ transient detection. While very close to GRU in time efficiency, its more complex gating mechanism results in slightly longer computation times, without a significant performance advantage. Third, standard Transformer requires significantly more computation time at 708.8 s/epoch. Although transformers can be easily parallelized, their global self-attention mechanism and large parameter space demand much higher computational power. This increased time is not justified by any improvement in Ca^2+^ transient predictability, which actually suffers due to interruptions from distal frames when global attention is used in decision-making. Fourth, Local Transformer is the least time efficient, taking 1,744.4 s/epoch, almost 6× slower than GRU or LSTM, and 2.5× slower than the standard Transformer. This can be attributed to the increased complexity of managing localized attention windows and maintaining context between them. Additionally, it loses parallelization efficiency when processing the entire window. However, this increased computation time is compensated by improved predictability, as attention is focused more effectively on local frames, leading to better decision-making. In conclusion, RNNs outperform transformers in both time efficiency and predictability for Ca^2+^ transient detection. Between two RNN variants, GRU offers a better performance in both time efficiency and predictability, although LSTM remains a viable alternative. When the GRU performance is challenged by dataset size, computational resources, and the complexity of the transient dynamics, other models (e.g., LSTM) could be explored directly in the CalTrig.

### Integrative visual exploration

The synchronization of Ca^2+^ imaging, cellular footprint, behavioral tracking, and Ca^2+^ transient trace windows, adjustable via a time bar, allows for a comprehensive and temporally aligned visualization of neuronal activity and behavior. This integration of multiple data streams provides several key benefits for neuroscience research. First, enhanced understanding of brain–behavior relationships: By synchronizing Ca^2+^ traces with behavioral data, researchers can directly observe how specific neuronal populations respond during behaviors or external stimuli. This real-time insight deepens our understanding of how brain activity drives behavior, such as the timing of Ca^2+^ transients relative to actions like lever presses. Second, technical flexibility and real-time adjustments: The adjustable time window enables researchers to zoom in on specific segments of data, closely examining how neural activity changes just before or after a behavioral event, which is critical for understanding causal relationships between stimuli and neural responses. Third, hypothesis proposing and testing: The integration of data streams facilitates hypothesis generation, which can be rapidly tested by determining the relationships between neural signals and behaviors. Researchers can observe whether certain Ca^2+^ transients precede or follow behavioral events, helping to refine hypotheses in real time. Fourth, visualization of micro-network dynamics: Viewing neural footprints while tracking behavior enables the identification of micro-networks at the neuronal level, distinct from traditional brain region-level network, during specific tasks or stimuli. This is critical for understanding neuronal network dynamics in healthy and diseased states, such as in learning, memory, or addiction studies. Fifth, categorization of neurons: Visualizing Ca^2+^ transients enable clustering neurons by activity patterns, revealing subpopulations and enhancing understanding of neural network heterogeneity in health and disease. Sixth, facilitating longitudinal studies: In long-term studies, where animals undergo repeated trials, integrative visualization tools allow researchers to track changes in both neuronal activity and behavior over time, aiding in the study of neuroplasticity and how neuronal responses evolve with experience. Seventh, improved data interpretation and collaboration: Simultaneous visualization of multiple data layers simplifies analysis, reveals patterns, and enhances cross-disciplinary communication. In conclusion, integrative visual exploration enables deeper exploration of brain function, supports hypothesis generation about neuronal mechanisms, and opens new avenues for understanding neurological disorders and developing treatments.

### Featured value of CalTrig

The usability of the CalTrig tool is highlighted by its integrated visual experience, which provides users with a comprehensive view of Ca^2+^ images, neuron footprints, and Ca^2+^ transient traces in one unified interface. The tool features multilayered toolboxes that cater to various analytical needs, allowing users to work efficiently at both the cellular and Ca^2+^ transient levels. Manual identification of Ca^2+^ transients is made efficient with features such as keyboard shortcuts (ASDF keys), hide/show traces, and unified scales for transient identification. This allows users to manually identify transients in ∼1–2 min per 15 min Ca^2+^ trace. Furthermore, manual identification can be assisted by autoidentification processes based on kinetic parameters or pre-established machine learning models. CalTrig is a self-looped tool, designed for continuous functional improvement, ensuring it remains adaptable and upgradable.

In terms of accessibility, CalTrig is a GUI-based open-source tool, making it readily shareable and open for further updates. It supports all CNMF or CNMF-E-processed imaging data and operates with limited computing demands, allowing for efficient performance even on less powerful systems. The GUI of CalTrig is designed to be user-friendly, ensuring that non-programmers can easily interact with it through intuitive buttons, menus, and forms. The tool presents a professional appearance, which enhances its suitability for presentations, collaborations, and publications. Real-time feedback is provided during parameter adjustments in Ca^2+^ transient identification, allowing users to fine-tune the process interactively. CalTrig can be downloaded as an independent application that runs in a Python environment, meaning users do not need additional software installations. Additionally, the tool is highly customizable, allowing users to tailor it to specific workflows and improving productivity for different use cases. Its expandability ensures that it can accommodate future updates and feature integrations.

Our research demonstrates that the GRU model provides high predictability when applied to testing cells from the same or different session stages, across various training days, brain regions, and mice. The GRU model has proven to be efficient in training, as shown in Figure 15, even with a limited number of cells (Figs. 18–20), and the “ground truth” of Ca^2+^ transients can be established with relative ease, as detailed in the Methods and Results sections. This makes it highly feasible to extend testing to longer training sessions, such as the 6 h sessions commonly used in studies on learning, memory, motivation, and addiction, or across broader time spans covering days, weeks, or months. The tool we developed here will be instrumental in evaluating the feasibility of in vivo Ca^2+^ recordings during extended sessions over prolonged recording periods.

The high predictability of the GRU model in detecting Ca^2+^ transients also indicates that the basic properties of Ca^2+^ transients, such as rise slope, amplitude, and signal-to-noise ratio, remained stable during 1 h recording sessions, across five recording days, and across different brain regions in several mice. Notably, the neurons detected in two brain regions, M2 and mPFC, are most likely pyramidal neurons, which may explain the model's high level of expandability. A future direction for research could be to test the expandability of the model to different types of neurons, e.g., interneurons.

### Limitations and future directions

There are several areas which the current study has not yet explored. For instance, the trained machine learning model has not been tested across different animal lines or species, such as from mice to rats. Additionally, while this study used GCaMP8f as the Ca^2+^ indicator, comparing its dynamics and fluorescent persistence with other genetically encoded Ca^2+^ indicators could provide valuable insights. Detecting Ca^2+^ transients is often just the starting point; challenges remain in selecting appropriate time windows for longitudinal analyses and linking behavior-associated transients to specific brain functions. Another intriguing area is the influence of neuronal spatial distribution on brain function, particularly under different physiological or pathological conditions. Our ongoing tool development aims to integrate these aspects into CalTrig, advancing the understanding of Ca^2+^ transients in brain output coding.

Classification of Ca^2+^ transients was performed using denoised and demixed calcium traces (“C” output) from the CNMF process implemented in Minian. Consequently, applying the provided machine learning models directly may limit their effectiveness to data processed using CNMF or similar algorithms. Performance could vary when utilizing calcium traces obtained from alternative signal extraction methods. However, if a new dataset obtained from non-CNMF processes, the user could still try to integrate it into the GUI. We recommend to look at each of the signals and see, which ones adhere most closely to the CNMF data structures. If there is no corresponding signal, then user may try to generate arrays of the same shape as the CNMF data. However, this could lead to some issues with a few of the features in the GUI. At the very least, the user should have the equivalent A, C, DFF, and a video file. If only a single video file is available, then the user could copy it for the other expected video files. We encourage users to open issues on the GitHub page.

## Synthesis

Reviewing Editor: Sam Golden, The University of Washington Decisions are customarily a result of the Reviewing Editor and the peer reviewers coming together and discussing their recommendations until a consensus is reached. When revisions are invited, a fact-based synthesis statement explaining their decision and outlining what is needed to prepare a revision will be listed below. The following reviewer(s) agreed to reveal their identity: Charles Zhou. Note: If this manuscript was transferred from JNeurosci and a decision was made to accept the manuscript without peer review, a brief statement to this effect will instead be what is listed below.

Dear Authors,

Your manuscript has been reviewed by two experts in the field. While both reviewers found the manuscript to be of interest, they have provided the following comments which must be addressed prior to eventual publication.

Reviewer 1:

Lange et al. introduce a new, user friendly, open-source graphical user interface tool (CalTrig) designed to address the challenges posed when analyzing calcium (Ca2+) transient data obtained by use of miniScopes in freely-behaving rodents. By integrating outputs from MiniAn (an open-source miniScope analysis pipeline), this new tool facilitates automatic identification of Ca2+ transients and their relation to specific operant behaviors. The authors evaluated four machine learning models (GRU, LSTM, Transformer, and Local Transformer) for Ca2+ transient detection. Ca2+ transient events from over 200 pyramidal neurons in motor (M2) and frontal cortices were used to compare the named machine learning models. They indicate that the GRU model offers the highest predictability and computational efficiency, achieving stable performance across training sessions, different animals, and brain regions.

This manuscript presents a well-developed, novel computational tool that significantly improves Ca2+ transient detection and analysis in freely-moving animals. The combination of machine learning, graphical use interface-based accessibility, and integration with behavioral tracking makes it a valuable contribution to neuroscience research. The combination of manual, parameter-based, and machine learning-based detection methods makes this tool flexible for different experimental needs. In particular, the application of machine learning models in Ca2+ transient analysis in freely-moving rodents is highly innovative and impactful. As someone involved in the painstaking endeavor of analyzing large amounts of data from miniScope recordings and behavioral tracking, I personally experienced the enormous challenges of this almost unsurmountable task, a possible reason why more neuroscientists are not using miniScopes. I wish this innovative tool had been available some years ago. Having at our disposal a reliable machine learning program for automatic detection of Ca2+ transients would have been particularly advantageous. Overall, this is a well-crafted and dense manuscript. I just have a few minor suggestions:

1. A full list of abbreviations would help the reader follow the different models.

2. While the sections on Ca2+ transient detection and analysis are very detailed, the association between Ca2+ transients and behavior seems underdeveloped. For example, how are the acquisition temporal parameters (e.g., frequency) from the behavioral videos time-locked with the Ca2+ transients during an operant behavior? Considering the long time lag (decay time) of Ca2+ transients, how efficiently will the program correlate them with a fast operant response such as a lever press?

3. The authors need to clarify in more detail how CalTrig differs from existing tools like CalmAn and Suite2P in specific applications, as this will enhance its positioning as a novel contribution.

4. While the manuscript is well-organized, consider summarizing key results in the Introduction or Discussion to immediately highlight the major findings.

5. In "Author Dedications" did the authors mean "Author Contributions"?

Reviewer 2:

Software Comments: Since the primary showcase of this paper is the GUI and underlying software, I believe it is important for the reviewers to evaluate the software with sample data. I was able to install the software in a relatively straightforward manner; however, the authors do not provide the reviewers nor potential users with sample data to test the pipeline. It would be great for authors to include sample data in the Github repository (or a cloud hosting service) that enables users to run and test the entire pipeline

The goal of this report is to provide the calcium imaging community with a novel analytical resource that inputs extracted calcium traces and performs post-processing in the form of visualization of calcium traces and calcium transient identification. The calcium transient detection model proposed in this manuscript is novel, generalizable, and promising. This manuscript indeed addresses a major gap in the resource literature and provides a great foundation (in terms of GUI, calcium transient detection, and conceptual framework); however, some important work remains to improve the manuscript and codebase clarity accessibility.

Major Concerns

1. Since the primary showcase of this paper is the GUI and underlying software, I believe it is important for the reviewers to evaluate the software with sample data. I was able to install the software in a relatively straightforward manner; however, the authors do not provide the reviewers nor potential users with sample data to test the pipeline. It would be great for authors to include sample data in the Github repository (or a cloud hosting service) that enables users to run and test the entire pipeline

2. The Github docs page does a good job of orienting users to the data format required for running the pipeline. There are a few improvements I suggest making to the doc:

a. In the "Before you Start" section, please provide more detail about the config file (e.g. where the file needs to be saved) and please also provide an example for the json file that, if I understand correctly, should inform of multi-session analysis.

b. For the overview section, please indicate which files are compatible with loading (or indicate which files in general need to be selected).

c. For the overview section, please state how one can open the GUI (ie. execute "python main.py" in anaconda when in the repository directory and with the relevant environment activated).

3. "Evaluation of CalV2N performance" section: The authors state that their motivation for this project was to provide a platform for evaluating CalV2N outputs using metrics such as rise time, peak amplitude, inter-transient intervals. However this section requires further elaboration. For example, it would be great to either show how these metrics can distinguish between poor and high quality detected cells, or at the very least reference other papers that educate on how to specifically use these metrics for identifying cells to reject. Further, I realized that Fig S9 and S10 show these metrics for analyzed cells in a spreadsheet format - I would somehow make a reference to these data in this section. Figure 3 could also be a section in the manuscript that this topic can be illustrated and elaborated on as authors provide a flow chart for quality control of CalV2N data, but no suggestions or insights on how to use the metrics/visualization to inform decisions.

4. I'm glad the authors include a limitations section; however, I believe there is an additional limitation that should be stated more clearly in the discussion: the authors utilize the "C" output of CaImAn/CNMF for training and testing the calcium transient classification model. This "C" data is the denoised and demixed calcium trace for neurons estimated by the CNMF algorithm. Importantly, this output is specific to the CNMF algorithm and using data from other algorithms may not perform as well when classifying new data.

5. "Applications for manual identification" section: This section was confusing to parse through because the title seems to be about manual identification of calcium transients, but the content pertains to automatic detection using a machine learning model. Further, I believe this section would benefit greatly from a figure illustrating each step. For example, the text detailing the first step indicates the user should label the calcium transient at the beginning stage of the project - a figure would be helpful to show the user where in the software the trace can be labelled for subsequent automatic detection.

6. The authors do a great job thoroughly testing the model across different datasets across time and across brain regions (Fig. 6-9). I would like to see more raw data showing the TP, FP, FN occurrences to get a sense of what transients the model is performing well or poorly on (for each model type - Fig 6-9). Fig. S8 shows a single snippit, but is not extensive nor annotated fully.

7. Could the authors please comment on how the calcium transient classification model performs with transients that are very closely grouped together or ride on top of eachother (take for example a transient that occurs before the previous transient fully decays, let's say 1 second between peaks).

Minor Concerns

1. Introduction - "Additionally, there are no efficient and reliable methods for identifying Ca²⁺ transients." : I understand the authors' goal with this statement; however spike deconvolution methods (Friedrich et al., 2017; Pachitariu et al., 2018) are technically calcium transient detectors. Perhaps a more accurate statement is that there are no efficient and reliable methods for identifying miniscope Ca²⁺ transients.

2. Supplementary snapshots are low resolution and sometimes difficult to view the smaller text. Please add higher resolution images.

3. Fig S3. The legend states the verified cells are marked by yellow; however the figure only shows a window of green highlighted focused cells. Please include the projection image/contour overlay from the GUI.

4. In 2.3.2, it seems like there is missing text for "Quality evaluation of CMNF"; also CMNF is a typo.

5. "Applications for manual identification" section: The GRU model is referred to in this section; however, the acronym nor an explanation of it is provided prior to this section. Please introduce and explain this model beforehand.

6. 2.3.4.2. "Parameter-based automatic identification": I believe a few sentences at the beginning of this section would orient readers and inform them what the importance of these 3 following parameters are.

7. Suggestion: A 200 sample segment length (~6.6 seconds) might be short considering calcium transients in the authors' data and in general can last longer than 10-15 seconds. Future tweaking of the model could include longer segment lengths

8. Please elaborate more in Fig. 5 legend how the model is structured as well as the format of data input and output.

9. Fig 7-9: Figure/legend needs to indicate which model was used.

10. Could the authors please specify what units (neurons/animals) and number of samples were used in the statistical tests for Figures 7-9? For example for 9L,M how many neurons from how many different animals were considered in the ANOVA.

Other

1. in vivo should be italicized (eg. in the introduction)

2. Methods (2.1.2): "tAAV1-Syn-jGCaMP8f-WPRE" I think the t is a typo

3. There is a case of "CalS2N" typo

## Author Response

We appreciate the positive comments from the Reviewers, including "a well-developed, novel computational tool that significantly improves Ca2+ transient detection ...", "a valuable contribution to neuroscience research", "flexible for different experimental needs", "highly innovative and impactful", "a well-crafted and dense manuscript", "include a limitations section", "a great job thoroughly testing the model across different datasets across time and across brain regions", etc. We also appreciate the reviewers' critiques, which we believe have significantly contributed to improving the quality and readability of the manuscript. Our replies to each point raised in the critique are listed below. Updates in the text are highlighted in blue in the manuscript.

R1Q1: A full list of abbreviations would help the reader follow the different models.

Reply: A table has been added at the end of the Introduction section to summarize key aspects of the study:

Key Abbreviations are listed in Table 1.

R1Q2: While the sections on Ca2+ transient detection and analysis are very detailed, the association between Ca2+ transients and behavior seems underdeveloped. For example, how are the acquisition temporal parameters (e.g., frequency) from the behavioral videos time-locked with the Ca2+ transients during an operant behavior? Considering the long time lag (decay time) of Ca2+ transients, how efficiently will the program correlate them with a fast operant response such as a lever press? Reply: Although our primary goal during CalTrig development was to evaluate CalV2N output quality and reliably, as well as efficiently identifying Ca2+ transients, the behavioral video and the corresponding behavioral data, for example the lever presses and the drug delivery in the intravenous model used in our team, can be loaded to CalTrig. Ca2+ traces can be segmented by any of these behavioral readouts. CalTrig can synchronize Ca2+ imaging, cellular footprint, behavioral tracking, and Ca2+ transient trace windows, which are adjustable via a time bar, allowing for a comprehensive and temporally aligned visualization of neuronal activity and behavior. This integration of multiple data streams provides several key benefits for neuroscience research. First, enhanced understanding of brain-behavior relationships: By synchronizing Ca2+ traces with behavioral data, researchers can directly observe how specific neuronal populations respond during behaviors or external stimuli. This real-time insight deepens our understanding of how brain activity drives behavior, such as the timing of Ca2+ transients relative to actions like lever presses. Second, technical flexibility and real-time adjustments: The adjustable time window enables researchers to zoom in on specific segments of data, closely examining how neural activity changes just before or after a behavioral event, which is critical for understanding causal relationships between stimuli and neural responses. Third, hypothesis proposing and testing: The integration of data streams facilitates hypothesis generation, which can be rapidly tested by determining the relationships between neural signals and behaviors. Researchers can observe whether certain Ca2+ transients precede or follow behavioral events, helping to refine hypotheses in real-time. This information is available in the updated manuscript.

R1Q3: The authors need to clarify in more detail how CalTrig differs from existing tools like CalmAn and Suite2P in specific applications, as this will enhance its positioning as a novel contribution.

Reply: CalmAn and Suite2P are both representative CalV2N tools. In the first paragraph of the INTRODUCTION, we outline the functional features of existing CalV2N tools, including CalmAn and Suite2P. However, considerable challenges remain after Ca2+ imaging data are processed by these CalV2N tools, such as the lack of synchronized visualization for multiple data streams, inconsistent results due to global parameter application, and uncertainty in verifying detected cells and Ca2+ traces. These challenges are detailed in the second paragraph of the INTRODUCTION. To address these post-CalV2N limitations, we developed the Ca2+ Transient Identifier GUI (CalTrig), a tool designed to verify and further process data from CalV2N tools, enhancing the accuracy and reliability of Ca2+ signal analysis.

R1Q4: While the manuscript is well-organized, consider summarizing key results in the Introduction or Discussion to immediately highlight the major findings.

Reply: This study aims to develop a tool that addresses a critical gap in processing miniScope Ca2+ imaging data. In the Discussion, we summarize the key advantages of this tool, including usability, accessibility, and predictability in 4.3. Featured Value of CalTrig. Additionally, we outline its limitations and potential improvements in 4.4 Limitations and Future Directions. We believe these sections provide a comprehensive and balanced summary of the CalTrig.

R1Q5: In "Author Dedications" did the authors mean "Author Contributions"? Reply: We have updated "Author Dedications" as "Author Contributions".

Reviewer 2:

R2Q1: Since the primary showcase of this paper is the GUI and underlying software, I believe it is important for the reviewers to evaluate the software with sample data. I was able to install the software in a relatively straightforward manner; however, the authors do not provide the reviewers nor potential users with sample data to test the pipeline. It would be great for authors to include sample data in the Github repository (or a cloud hosting service) that enables users to run and test the entire pipeline Reply: We've included https://drive.google.com/drive/folders/143QBRTsuf5SMr1bU-a6rqJvcvzux7Z9r with a link available in the Overview section of the documentation, as well as the main Github page. See the related information by clicking this link (https://calcium-transient-analysis.readthedocs.io/en/latest/Overview.html#demo-data) R2Q2: The Github docs page does a good job of orienting users to the data format required for running the pipeline. There are a few improvements I suggest making to the doc:

R2Q2a: In the "Before you Start" section, please provide more detail about the config file (e.g. where the file needs to be saved) and please also provide an example for the json file that, if I understand correctly, should inform of multi-session analysis.

Reply: The config file can be saved anywhere convenient for the user, as the GUI contains a file searching interface to select the desired config file. This has been clarified in the https://calcium-transient-analysis.readthedocs.io/en/latest/Before_you_start.html#creating-the-config-file. An example of the json file has been added to the Overview Section (see more details by clicking "https://calcium-transient-analysis.readthedocs.io/en/latest/Overview.html#main-window"). The related updates are also pasted below for your reference. "The final step is to create a config.ini file that will tell the GUI where to find the necessary data. This file can be saved anywhere convenient for the user as the GUI contains a file picker to select the desired config file. For ease of use, it is recommended to save the config file near the CalTrig folder. Below is a template that you can adjust to your needs:" R2Q2b: For the overview section, please indicate which files are compatible with loading (or indicate which files in general need to be selected).

Reply: The "Minian Project" and "Non-Minian CNMF Projects" in the [Before you Start] (https://calcium-transient-analysis.readthedocs.io/en/latest/Before_you_start.html) section details the exact files and format that are needed in order to run CalTrig. Once the necessary data has been allocated to the corresponding "data", "videos" and "timestamps" locations, the created config files will point to these locations once loaded in the GUI.

Once the User starts the GUI and selects File->Load Data, they then select the config file as it contains all the necessary information for a single session. The overview section has been updated to clarify this. See more details by clicking "https://calcium-transient-analysis.readthedocs.io/en/latest/Overview.html#main-window". The related updates are also pasted below, including the screenshots of "Non-Minian Projects" and "Minian Projects" for your reference. "To load in a specific dataset, click File -> Load Data then proceed to select the config file you have created in https://calcium-transient-analysis.readthedocs.io/en/latest/Before_you_start.html#creating-the-config-file." R2Q2c: For the overview section, please state how one can open the GUI (ie. execute "python main.py" in anaconda when in the repository directory and with the relevant environment activated).

Reply: The Installation section has been expanded with step-by-step details to address this comment. Please click the link here to reach the corresponding section: https://calcium-transient-analysis.readthedocs.io/en/latest/Overview.html#installation. A screenshot of the related information is pasted here for your convenience.

R2Q3: "Evaluation of CalV2N performance" section: The authors state that their motivation for this project was to provide a platform for evaluating CalV2N outputs using metrics such as rise time, peak amplitude, inter-transient intervals. However this section requires further elaboration. For example, it would be great to either show how these metrics can distinguish between poor and high quality detected cells, or at the very least reference other papers that educate on how to specifically use these metrics for identifying cells to reject. Further, I realized that Fig S9 and S10 show these metrics for analyzed cells in a spreadsheet format - I would somehow make a reference to these data in this section. Figure 3 could also be a section in the manuscript that this topic can be illustrated and elaborated on as authors provide a flow chart for quality control of CalV2N data, but no suggestions or insights on how to use the metrics/visualization to inform decisions.

Reply: We added Figure 9 to illustrate example Ca2+ traces and their dynamic values from accepted and rejected cells.

Figures. S9, S10, which are Figs. 16 and 17 in the updated version, have been presented with a better resolution:

The section of "2.3.3.Cell verification and CalV2N evaluation" directly corresponds to Fig. 5 (corresponding to Fig. 3 in the previous version), detailing the steps in using CalTrig to validate and filter cells identified by CalV2N. In addition to this section in the main text, we have also added more details to the figure legend for Fig. 5 to further clarify these steps. Below is the newly developed Fig. 5 legend for your reference:

This flowchart illustrates the workflow for validating and filtering cells detected by CalV2N using CalTrig. The process begins with in vivo calcium imaging data collection, followed by CalV2N analysis, which performs cell identification and calcium transient extraction. To assess the quality of detected cells, a visual exploration tool is used, allowing interactive inspection of calcium video, footprint visualization, behavioral video, and temporal traces, including raw signal, C, S, ΔF/F, noise, and signal-to-noise ratio.

Users determine whether to accept or reject a detected cell based on visual confirmation of its footprint, calcium image, and calcium transients. If a cell is accepted, it proceeds to further processing for transient event confirmation. If rejected, the reasons for rejection are evaluated, which may include poor signal quality, suspicious footprint, or statistical outliers. The rejection process also considers whether the issue stems from suboptimal CalV2N performance, in which case parameters for calcium transient extraction can be optimized. If CalV2N errors persist, improvements in raw imaging data quality should be considered. See more details in "2.3.3.Cell verification and CalV2N evaluation" R2Q4: I'm glad the authors include a limitations section; however, I believe there is an additional limitation that should be stated more clearly in the discussion: the authors utilize the "C" output of CaImAn/CNMF for training and testing the calcium transient classification model. This "C" data is the denoised and demixed calcium trace for neurons estimated by the CNMF algorithm. Importantly, this output is specific to the CNMF algorithm and using data from other algorithms may not perform as well when classifying new data. (Df/f) Reply: This is a valid observation; however, we would like to clarify that the "C" output has been taken from the Minian processing and CaImAn was only utilized to calculate "Df/f". We have updated our manuscript with the following section which is also available in GUI section "What About Non-CNMF signals" in "Before you start" (https://calcium-transient-analysis.readthedocs.io/en/latest/Before_you_start.html) "Classification of Ca2+ transients was performed using denoised and demixed calcium traces ("C" output) from the CNMF process implemented in Minian. Consequently, applying the provided machine learning models directly may limit their effectiveness to data processed using CNMF or similar algorithms. Performance could vary when utilizing calcium traces obtained from alternative signal extraction methods. However, if a new dataset is obtained from non-CNMF processes, you could still try to integrate it into the GUI. We recommend to look at each of the signals and see which ones adhere most closely to the CNMF data structures. If you don't have a corresponding signal, then you could try to generate 0 arrays of the same shape as the CNMF data. However, this could lead to some issues with a few of the features in the GUI. At the very least, you should have the equivalent A, C, DFF and a video file. If you only have a single video file, then you could copy it for the other expected video files. All of this is theoretical and hasn't been tested before. In case of any problems arise, please open an issue on the https://github.com/cci-MaLab/Calcium-Transient-Analysis/issues page." R2Q5: "Applications for manual identification" section: This section was confusing to parse through because the title seems to be about manual identification of calcium transients, but the content pertains to automatic detection using a machine learning model. Further, I believe this section would benefit greatly from a figure illustrating each step. For example, the text detailing the first step indicates the user should label the calcium transient at the beginning stage of the project - a figure would be helpful to show the user where in the software the trace can be labelled for subsequent automatic detection.

Reply: We appreciate the constructive suggestion to illustrate the multiple applications of manual identification in a figure. We have updated Fig. 7 to better represent the procedures of Ca2+ transient identification. Circled numbers (1-4) now mark the corresponding steps for the four applications discussed in the main text. Additionally, the figure legend and the related text have been revised to reflect these modifications.

R2Q6: The authors do a great job thoroughly testing the model across different datasets across time and across brain regions (Fig. 6-9). I would like to see more raw data showing the TP, FP, FN occurrences to get a sense of what transients the model is performing well or poorly on (for each model type - Fig 6-9). Fig. S8 shows a single snippit, but is not extensive nor annotated fully.

Reply: All the raw data of TP, TN, FP, FN are available in the Appendix section of our GUI documentation, as shown in the screenshot of the webpage.

In the webpage, we provided a following link to download the Excel file, "TN TP FN FP Raw data.xlsx", which has been clarified in the main text too. https://github.com/cci-MaLab/Calcium-Transient-Analysis/raw/refs/heads/main/ml_training/caltrig_results/TN%20TP%20FN%20FP%20Raw%20data.xlsx R2Q7: Could the authors please comment on how the calcium transient classification model performs with transients that are very closely grouped together or ride on top of eachother (take for example a transient that occurs before the previous transient fully decays, let's say 1 second between peaks) Reply: Thank you for this insightful critique. When calcium transients occur in rapid succession, accurately determining whether a neuron fired once or twice can be challenging. The decision on two potential adjacent Ca2+ peaks with a small Inter-Transient Interval (ITI) has been strictly made based on the pre-set minimum ITI in Parameter-based automatic identification. ITI measures the frame distance between the initial frames of two adjacent Ca2+ transients. If this distance is shorter than the pre-set threshold, the transients are merged, with the lower peak combined into the higher peak. However, a peak occurring within the prolonged decay slope of a preceding transient but exceeding the interval threshold is counted as an independent event. Its amplitude is calculated as the difference between its peak ΔF/F and the ΔF/F at its rising phase onset. This criterion was consistently applied when establishing the ground truth dataset for training our machine learning models. Notably, our machine learning model, particularly the GRU model trained with data from more than 10 cells, successfully identified and classified each transient as a distinct event, which was evidenced by the values of macro F1 beyond 0.95 in most cases. Specifically, we have used 50 frames at 30 Hz sampling rate, i.e., 1.67 sec, as the minimal ITI. This number can be directly adjusted in the window of "Trace toolbox" on GUI interface.

R2Q8: (minor) Introduction - "Additionally, there are no efficient and reliable methods for identifying Ca2+ transients." : I understand the authors' goal with this statement; however spike deconvolution methods (Friedrich et al., 2017; Pachitariu et al., 2018) are technically calcium transient detectors. Perhaps a more accurate statement is that there are no efficient and reliable methods for identifying miniscope Ca2+ transients.

Reply: Thank you for your thoughtful comment. We truly agree and it has been updated as suggested.

R2Q9: (minor) Supplementary snapshots are low resolution and sometimes difficult to view the smaller text. Please add higher resolution images.

Reply: The image resolution of the screenshots in the these figures has been improved as suggested by this reviewer.

R2Q10: (minor) Fig S3. The legend states the verified cells are marked by yellow; however the figure only shows a window of green highlighted focused cells. Please include the projection image/contour overlay from the GUI.

Reply: Thank you for pointing out this discrepancy. It has been corrected to: "The verified cells are highlighted in green." In Fig. 6 (corresponding to Fig. S3 in the previous submission) R2Q11: (minor) In 2.3.2, it seems like there is missing text for "Quality evaluation of CMNF"; also CMNF is a typo.

Reply: We have added a brief descript of "Quality evaluation of CNMF analysis" in 2.3,2, "CalTrig allows user to verify detected cells, manually identify missing cells, and refine transient detection (see details in section 2.3.3)." The typo has been corrected.

R2Q12: (minor) "Applications for manual identification" section: The GRU model is referred to in this section; however, the acronym nor an explanation of it is provided prior to this section. Please introduce and explain this model beforehand.

Reply: To prevent potential confusion from introducing GRU too early, we have updated this section without specifying the GRU model. Instead, we discuss the third application for manual identification, focusing on post-processing data from machine learning models in general. Details on GRU are provided in a later section (i.e., 2.3.4.3 Detection using Machine Learning Model).

R2Q13: (minor) 2.3.4.2. "Parameter-based automatic identification": I believe a few sentences at the beginning of this section would orient readers and inform them what the importance of these 3 following parameters are.

Reply: We have added the following sentences before elaborating the methodological details of the parameter-based automatic identification: "Although the variability in Ca2+ transient characteristics is well recognized, the fundamental dynamic parameters of Ca2+ transients should fall within a certain range. Thus, establishing key dynamic parameters as a digital filter can streamline the Ca2+ transient identification process, improving both accuracy and efficiency." R2Q14: (minor) Suggestion: A 200 sample segment length (~6.6 seconds) might be short considering calcium transients in the authors' data and in general can last longer than 10-15 seconds. Future tweaking of the model could include longer segment lengths Reply: Thank you for this insightful consideration. We have added the following comment after the 200-frame size in the main text: "Considering the slow dynamics of Ca2+ transients, which may last 10-15 seconds, increasing the segment length could be explored in future optimizations to further improve transient detection." R2Q15: (minor) Please elaborate more in Fig. 5 legend how the model is structured as well as the format of data input and output.

Reply: We have updated the legend for Fig. 13 (corresponding to Fig. 5 in the previous submission) to include more details.

Figure 13. Flowchart demonstrating Ca2+transient validation via machine learning models.

A, Application of RNNs in predicting Ca2+ transients. The input consists of the C signal and ΔF/F traces. A sequence of GRUs or LSTM modules processes the temporal dynamics of the Ca2+ transient. The network includes multiple stacked layers, with fully connected layers (FNN) at the final stage to produce the predicted Ca2+ transient.

B, Application of Transformer models for predicting Ca2+ transients. The input is first expanded through a dimensional transformation step, followed by repeated attention-based self-mapping in either a standard Transformer or a local Transformer architecture. The processed features are then passed through a feedforward network (FNN) to generate the predicted Ca2+ transient.

C, D, Diagrams showing the internal operations of two variants of RNN, i.e., GRU module (C) and LST module (D). The GRU uses two gates: the update gate, which controls the amount of information passed to the next step, and the reset gate, which determines how much of the previous information to forget. These gates modify the hidden state H(t) at the current time step based on the input X(t) and the previous hidden state H(t−1), leading to a new hidden state H(t). The LSTM module architecture is depicted, showcasing its cell structure. It uses three gates: forget, input, and output gates to control the flow of information. The input X(t), the previous cell state C(t−1), and the previous hidden state H(t−1) are processed through these gates to update the cell state C(t) and produce the new hidden state H(t).

E, F, Diagram showing the Transform (E) and local Transformer (F) mechanisms. Multiple columns represent the same set of data. Non-translucent colored regions highlight the specific values being processed, while translucent colors represent comparison values within the self-attention mechanism. No color denotes values excluded from evaluation, enforcing global attention to all time points. Local Transformer mechanism. Unlike the standard Transformer, the local Transformer focuses only on a subset of adjacent time points, restricting the attention scope to a local temporal window. This reduces computational complexity while maintaining essential local dependencies in Ca2+ transient predictions.

Data input format: The models receive sequential time-series data of C signals and ΔF/F traces as input.

Data output format: The models predict Ca2+ transients by predicting for each timestep whether it is part of a transient or not (1 for transient otherwise 0), a consecutive, unbroken sequence of 1s is considered to be part of a single transient event, this prediction is saved to the E data structure.

FNN, Feedforward neural network.

R2Q16: (minor) Fig 7-9: Figure/legend needs to indicate which model was used.

Reply: The GRU model was used to obtain the data presented in Figs. 18-20 (corresponding to Figs. 7-9 in the previous submission), which are specified in the corresponding figure legends.

R2Q17: (minor) Could the authors please specify what units (neurons/animals) and number of samples were used in the statistical tests for Figures 7-9? For example for 9L, M how many neurons from how many different animals were considered in the ANOVA.

Reply: These details have been added to the figure legends of Figs. 18-20 (corresponding to Figs. 7-9 in the previous submission).

R2Q18: (other) in vivo should be italicized (eg. in the introduction) Reply: We have italicized "in vivo" in the Introduction.

R2Q19: (other) Methods (2.1.2): "tAAV1-Syn-jGCaMP8f-WPRE" I think the t is a typo Reply: Thank you for pointing it out. The identified type has been corrected by deleting "t".

R2Q20: (other) There is a case of "CalS2N" typo Reply: Thank you for pointing it out. This typo has been corrected.
